# Explainable AI in Healthcare: A Holistic View of Technical Approaches, Regulatory Frameworks, Reliable Clinical Implementation Insights and Limitations

**DOI:** 10.3390/diagnostics16193107

**Published:** 2026-09-24

**Authors:** Guilherme Barbosa, Eduardo Carvalho, Dulce Oliveira, Ana Rita Moura, Ana Guerra, Francisca Pinheiro, Ricardo Correia, André Martins Dos Santos, Nilza Ramião, Miguel Mascarenhas

**Affiliations:** 1 Instituto de Ciência e Inovação em Engenharia Mecânica e Engenharia Industrial, Campus of the Faculty of Engineering, Universidade do Porto (FEUP), R. Dr. Roberto Frias 400, 4200-465 Porto, Portugalaguerra@inegi.up.pt (A.G.);; 2 BioGHP, Rua da Constituição, Nº 656, 1º, Sala 117, 4200-194 Porto, Portugalricardo@bioghp.com (R.C.);; 3 Faculty of Medicine, Universidade do Porto (FMUP), Alameda Prof. Hernâni Monteiro, 4200-319 Porto, Portugal; miguelmascarenhassaraiva@gmail.com; 4 World Gastroenterology Organisation (WGO) Porto Gastroenterology and Hepatology Training Center, Campus of Faculty of Medicine, Universidade do Porto (FMUP), Alameda Prof. Hernâni Monteiro, 4200-319 Porto, Portugal; 5 Department of Gastroenterology, São João University Hospital, Alameda Prof. Hernâni Monteiro, 4200-319 Porto, Portugal

**Keywords:** explainable artificial intelligence, large language models, deep learning, biomedical ethics, model bias, transparency

## Abstract

Opaque deep learning models in biomedical applications pose challenges related to bias, fairness, and regulatory compliance. This paper presents a narrative review of explainable artificial intelligence (XAI) in healthcare, specifically medical diagnosis, based on a targeted selection guided by a narrative review of recent literature, from 2019 to 2026, conducted on Scopus, Web of Science, and PubMed databases. Covered topics comprise model interpretability, including image-based models and multimodal models, with special focus on large language models (LLMs) as a hard-to-interpret system. The review situates XAI within key regulatory frameworks, including the European Union (EU) Artificial Intelligence (AI) Act, highlighting how explainability supports legal requirements for transparency, auditability, and accountability. It examines widely used XAI methods such as Shapley Additive Explanations (SHAP), Local Interpretable Model-Agnostic Explanations (LIME), attention visualization, neurosymbolic reasoning, and others. These techniques help identify bias, detect spurious correlations, and analyze hallucinations in LLM deployment. They present an effort to, within a feasible range, minimize these events and increase confidence in the predictions provided, without dispensing with clear and proper validation within the medical classification loop. While emphasizing the benefits of XAI, the review also addresses key limitations, including limited explanation fidelity, cognitive overload, and the risk of misleading interpretations. Overall, it provides a concise synthesis of how explainability intersects with ethics, regulation, and system design to support oversight and transparency in AI healthcare. Additionally, it provides suggestions for the incorporation of technical XAI methods within current legislation and guidelines to provide a robust framework for AI model deployment.

## 1. Introduction

Throughout our work, the following terms are employed with their respective meaning: explainability refers to how methods applied in AI provide explanations that are understandable by physicians; interpretability refers to how clear the inner functioning of the model is; transparency relates to how a process is clear, reproducible, and justified; trustworthiness refers to the performance of a model and how confident it is in its decisions; reliability refers to how accurate the model is; accountability refers to the degree to which every model’s decision is always traceable to a specific model version; and finally, fairness refers to the mitigation of bias towards specific subgroups in a model’s decision.

Artificial intelligence (AI) is rapidly transforming healthcare by providing advanced computational models that assist clinicians in diagnosis, treatment planning, and decision-making [1,2]. These models, ranging from image-based and multimodal systems to large language models (LLMs), offer unprecedented capabilities for analyzing complex biomedical data. However, as the sophistication of AI systems increases, so does the challenge of understanding and interpreting their decisions. Without sufficient transparency, AI outputs risk being perceived as “black boxes,” which limits trust and hinders clinical adoption.

A central response to this challenge lies in explainability: the ability to understand how and why a model produces a particular output, which is critical in healthcare settings, such as in medical diagnosis tasks. It enables clinicians and stakeholders to validate AI decisions, align them with ethical standards, and ensure compliance with evolving regulations, such as the European Union (EU) AI Act [3]. Moreover, explainable AI (XAI), when accurately and correctly applied, supports the principles of biomedical ethics, including beneficence, non-maleficence, autonomy, and justice, by promoting accountability and patient safety. Although incorrect explanations might seldom be misleading or manipulative, overall XAI contributes to the embodiment of these principles.

A real-world grounded enforcement of these principles and regulations requires proper technical guidance. This review provides a comprehensive examination of technical approaches to XAI, focusing on their applicability in healthcare. It examines data exploration techniques, intrinsic methods of model explainability, the trade-off between model complexity and interpretability, as well as model-agnostic, model-specific, and post hoc approaches. By synthesizing these strategies, the review highlights how explainability can bridge the gap between complex AI models and the transparency required for the practical, ethical, and regulatory requirements of modern medicine.

The combination of these explainability methods provides grounded decision-making for physicians, patients, and hospital management centers. All these stakeholders benefit from improved and transparent healthcare. This review provides an extended survey of technical approaches that implement ethical and legislative procedures for healthcare transparency through explainability in AI. Furthermore, the impact of these technical approaches on healthcare for each stakeholder is explained.

Physicians have the opportunity to provide better and more informed care for patients, in addition to streamlining the procedures in place. With increased trust in deep learning-automated models, healthcare pipelines can be streamlined and enhanced. Faster and more accurately grounded decision-making takes place, significantly improving patients’ lives and additionally reducing the burden on healthcare systems.

An example of how deep learning and AI can improve patient outcomes is presented by Shen et al. [4]: in a case study from Tongji Hospital in China, the implementation of LLMs to aid Alzheimer’s diagnosis has resulted in an improvement of 6% in diagnostic accuracy and an average reduction of 37 min in assessment time.

Hospital management similarly benefits from these inclusions, with the relief of the healthcare system burden. Cost savings and faster examinations provide better care for patients. Acosta et al. [5] demonstrate how the implementation of AI workflows reduced the reporting time from 573 to 435 s. This represents major time-saving in clinical care.

Commercial healthcare solutions are currently available in the market and constitute a large enterprise. Brainomix is an example of such a system. It presents an imaging application that detects signs of hypodensity and ischemia in CT brain scans. It presents real case studies for stroke prediction [6]. Bone-viewing technology [7] is used for fracture detection in radiographs. These systems point towards lesions, such as fractures, dislocations, or other anomalies. It indicates, with bounding boxes, the respective lesion location and type. Both of these enterprises were selected based on their market-ready availability.

These commercial applications signify how AI can be implemented in medicine. This improved and expedited care presents advantages in both clinical and economic settings. This benefits all stakeholders involved, including physicians, patients, and hospital management. However, the use of such operations is still relatively limited, and development opportunities often meet ethical or legal and regulatory barriers. For these products to be marketable within the EU, certifications from the *Conformité Européenne* (CE) mark are crucial. This imposes real regulatory barriers that, although certainly required, mitigate the propagation and implementation of this technology.

To obtain the CE Mark, Article 48 of the EU AI Act must be upheld. This Article provides a link between the CE Mark and the EU AI Act that ensures every medical device follows this framework. The EU AI Act does not prescribe specific explainability mechanisms such as saliency maps, heatmaps, SHAP, or neurosymbolic reasoning. Instead, it adopts a technology-neutral approach, requiring that high-risk AI systems provide sufficient transparency and information to enable deployers to interpret outputs, understand limitations, and exercise effective human oversight. Consequently, XAI may serve as a compliance-enabling technique, but specific XAI methods are not mandated by the regulation.

Specific guidelines determining what makes a solution, deep learning-based or not, interpretable, transparent, and mostly trustworthy and reliable are important steps for future directions. This review attempts to fill this gap by identifying technically valid models and explainability solutions that could even be incorporated into legislative procedures. Moreover, it constitutes a first step towards defining how a healthcare system can be interpretable, trustworthy, and transparent. The incorporation of explainability methods, together with the current legislative framework that ensures clinical validity, robustness testing, and continuous calibration, is a step forward. Although explainability is a required complement, it does not replace the current legislative procedures. This can hopefully increase the confidence of all stakeholders, physicians, patients, and hospital management in computer-aided clinical care. This intersection between technical aspects, ethics and legislation incorporates the novelty step into this review. Through this intersection, an enhancement to current legislation can be achieved.

Throughout this review, there is a summary of how impactful AI is in healthcare, encompassing image-based models, multimodal models, and LLMs. Afterwards, the need and relevance of explainability methods, transparency, trustworthiness, and compliance with ethical and regulatory requirements are defined. To conclude, a discussion describing the intersection between ethical, legal, and technical domains, jointly with possible future directions and guidelines, is presented.

In the current state of the art, multiple reviews present various perspectives regarding the incorporation of XAI methodologies within the healthcare system. Aravindkumar et al. [8] present multiple use cases that exemplify how methodologies such as SHAP, LIME, or attention visualization methods contribute towards transparency in healthcare and a standardization of XAI deployment methodologies. Shiddik et al. [9] present a systematic review of over 70 studies that include multiple models, from simpler AI methods to complex neural network approaches, and present a range of explainability techniques from SHAP and LIME to attention-based mechanisms. The trends of each approach are quantified, and gaps such as the lack of real-world validation, standardized approaches, and specialized metrics for interpretability are discussed. Zhang et al. [10] quantify what meaningful explainability is for each stakeholder within different clinical contexts and measure clinical utility and alignment with clinician confidence. Frasca et al. [11] establish a bridge between ethics and trust experienced by clinicians and patients. Agbo Eje et al. [12] describe the need for standardization of explainability metrics across studies, and point out the gap in existing legislative frameworks such as the Food and Drug Administration (FDA), General Data Protection Regulation (GDPR), and Health Insurance Portability and Accountability Act (HIPAA) for specific XAI methodology implementations. In the analyzed reviews, there is a clear gap related to the implementation of technical frameworks within EU legislation and an encompassing holistic review of ethical, legislative, and regulatory frameworks.

This review attempts to provide useful insights into XAI procedures in the developing EU legislation to fill an existing gap in the specific requirements for transparency within model deployment. Although some reviews mention the connection between regulation, AI, and healthcare, we provide a holistic view: from an ethics foundation that describes the core principles of modern biomedical research and medicine to the edification of the EU AI Act. We address the current limitations within this legislation and describe technical implementations with respective use cases to provide real-world examples. Our goal is to reframe how transparency is approached in the EU AI Act and suggest future steps in policy development.

## 2. Materials and Methods

This study presents a narrative review of the current implementation of AI in healthcare and the available explainability methods. The literature search covered publications from 2019 to the 20th of July 2026; foundational papers outside of this timeline that describe the initial theory behind a concept have been included. The search was conducted using Scopus, Web of Science, ScienceDirect, and PubMed databases. The search strategy was based on keywords such as ((“artificial intelligence” OR AI) AND healthcare (“explainable artificial intelligence” OR “explainable AI” OR XAI) AND healthcare). The field restrictions were limited to Title, Abstract, Keywords, and Full-text. To fully represent each study and use case, the clinical task was described, jointly with the performance metric and XAI method when justified.

The search was conducted in English; articles in other languages were excluded. Although the exclusion of papers from other languages may introduce some bias into this research, it also minimizes the risk of errors and misinterpretations that may occur in translation. During the screening process, original research articles and systematic and narrative reviews were all considered. The selection focused on work developed within EU territory, preferably with external validation incorporated into the study, and from validated sources such as peer-reviewed journals. Although these attributes were an advantage, papers without these requirements were also considered. As exclusion criteria, purely administrative systems and non-English papers were disregarded. This process was conducted by a single reviewer. To organize all the cited work, Zotero 9.0.6 software was used.

During the preparation of this manuscript, language-support tools (Microsoft Copilot with GPT-5 and Grammarly) were used to improve clarity, style of expression, literature identification, summarization, drafting, and data extraction. All intellectual contributions, analyses, and scientific interpretations are entirely the responsibility of the authors, who carefully reviewed and edited the text.

## 3. The Relevance of AI in Healthcare

AI is advancing rapidly in healthcare, with research output expanding at an unprecedented pace. As illustrated in Figure 1, the number of AI-related publications in healthcare has grown substantially in recent years, reflecting the field’s accelerating importance and research attention. Figure 1 presents the number of publications per year that appear on ScienceDirect with the keywords ((“artificial intelligence” OR AI) AND healthcare) for the AI in Healthcare category, and (((“explainable artificial intelligence” OR “explainable AI” OR XAI) AND healthcare), for the XAI in healthcare category. The terms were matched with Title, abstract or author-specified keywords. The search extraction was implemented on the 3rd of August of 2026. Within this domain, three primary modalities can be distinguished: traditional image-based models, multimodal models that integrate tabular and imaging data, and the more recent emergence of LLMs, which are increasingly assuming a central role in healthcare innovation and clinical support.

The most accurate models deployed in healthcare settings are deep learning-based and include purely image-based models, multimodal models, and LLMs. These models are opaque black boxes with very good results, and their increasing growth in healthcare justifies the need for transparency measures and explainable solutions. The performance of these models and relevant case studies are discussed in the following sections.

### 3.1. Image-Based Models

The integration of AI into healthcare, particularly within clinical decision-support systems for medical diagnosis, has advanced rapidly in recent years. Among its most prominent applications is medical image classification, which spans a wide range of modalities, including whole-slide imaging (WSI) [13], X-ray [14], positron emission tomography (PET) [15], magnetic resonance imaging (MRI) [16,17], ultrasound [18,19], computed tomography (CT) [15,20], and optical coherence tomography (OCT) [21,22,23,24], among others.

X-ray image analysis was one of the earliest applications of deep learning in healthcare. Chest X-rays (CXRs) are cost-effective and clinically informative, making them one of the most widespread diagnostic modalities. The abundance of curated datasets has provided favorable conditions for the development of AI-based diagnostic tools in this field [7]. Public repositories such as CheXpert [25] have enabled large-scale research on disease classification in CXRs. Yuan et al. [26] achieved state-of-the-art performance on CheXpert, with an area under the curve (AUC) of 0.93, where an AUC of 1 represents perfect discrimination, and 0.50 corresponds to random guessing. Rajaraman et al. [20], with a different dataset, demonstrated that AI models trained on CXRs can accurately classify pneumonia-related abnormalities, further illustrating the robustness and clinical potential of deep learning approaches in radiographic analysis.

WSI has emerged as a gold standard for histological digital imaging modality in both clinical diagnostics and research, particularly in pathology. The increasing availability of WSI data has accelerated the adoption of computer vision methods across diverse applications. Shi et al. [27] demonstrated that neuropathological tumor classification and proliferation prediction can be achieved through WSI analysis. Likewise, Poudel et al. [28] applied deep learning techniques to distinguish between high and low microsatellite instability (MSI), an accumulation of errors in the Deoxyribonucleic acid (DNA) sequence due to a failure in the mismatch repair system that leads to a variation in the length of short repetitive DNA sequences, designated as microsatellite. Their approach is based on a two-stage framework that includes WSI patch segmentation of cancerous tissue and classification of MSI or non-MSI status. MSI is a clinically relevant biomarker for cancer prognosis. Nan et al. [29] further highlighted the versatility of WSI by accurately categorizing five distinct types of skin lesions. Collectively, these studies highlight the increasing significance of WSI in AI-driven diagnostic pipelines.

Neuroimaging for Alzheimer’s disease (AD) has been advanced through initiatives such as the Alzheimer’s Disease Neuroimaging Initiative (ADNI) [30], which provides a rich repository of MRI and PET scans along with associated biomarkers. Building upon these resources, Alsaraira et al. [31] developed an MRI-based framework for detecting AD and mild cognitive impairment, achieving an AUC of 0.94. Integrating MRI and PET modalities offers even greater potential for disease classification, as presented by Suk et al. [32], who reported accuracies of 93.52% for AD diagnosis, demonstrating the relevance of such applications in this domain.

CT imaging has similarly benefited from AI-based solutions. Peters et al. [33] reported that computer-aided diagnosis improved mean nodule detection rates for both junior and senior radiology residents. More recently, He et al. [34] introduced the Versatile Imaging SegmenTation and Annotation model (VISTA3D), a neural network capable of automatically segmenting 3D CT scans across axial, coronal, and sagittal planes. This approach reduces processing time and enhances diagnostic efficiency in radiology workflows by incorporating multiple scans into a 3D input for direct zero-shot approaches.

Deep learning has also been applied to OCT, particularly for retinal pathology classification. In two separate studies, Rebolo et al. [21] and Barbosa et al. [22]—with more than 500 testing inputs and four distinct classes: retinal vein occlusion (RVO), diabetic macular edema (DME), Normal, and combined multiple pathologies—developed models capable of distinguishing DME from RVO, achieving classification accuracies exceeding 90% and an AUC above 0.9.

Ultrasound imaging represents another domain where AI shows significant promise. For instance, AI-based ultrasound methods have been employed to support the diagnosis of polycystic ovary syndrome [35], as well as to assess fetal health. Existing systems can identify fetal situs, display recommended anatomical views, evaluate brain development, and adjust Doppler measurements of cardiac output [36,37].

Beyond traditional medical imaging, AI-driven diagnostic tools are also being adapted for mobile applications. For example, smartphone-based models have been developed to assist in skin lesion diagnosis [38], thereby extending access to diagnostic resources and broadening the reach of healthcare.

Image-based models are normally developed on a convolutional neural network or transformer backbone that lacks directly interpretable decision pathways. These black box models require some extended explanation and reasoning to provide a transparent outcome. Explainability methods are key to providing such transparency.

### 3.2. Multimodal Models

Medical decisions are rarely based on a single source of information. Instead, they rely on a complex integration of multiple variables. To emulate this clinical reasoning and achieve optimal predictive performance, multimodal models have emerged. By incorporating diverse inputs, ranging from imaging data to tabular clinical records, these models more closely reflect the multifaceted nature of medical practice.

Inputs such as omics data, imaging studies, laboratory results, electronic health records (EHRs), vital signs, and information from wearable devices all provide complementary insights [39]. Their integration can reveal patterns that would remain hidden if considered in isolation. For example, combining data from proto-oncogenes (molecular pathways that regulate cell growth and, when mutated, contribute towards the uncontrolled cell growth that characterizes cancerous behavior) such as epidermal growth factor receptor—a protein in the cellular membrane that binds to growth factors, when exaggerated by fusion or mutations, causes the cell to multiply uncontrollably; anaplastic lymphoma kinase—a membrane receptor that aids in the development of the nervous system in early growth, when mutated, this receptor can lead to rapid cell growth and cancer; *ROS1*, rearranged during transfection (*RET*), *MET* and *Erb-B2 Receptor Tyrosine Kinase 2* (*ERBB2*)— these genes codifya different receptor of the tyrosine kinase protein, mutations in these genes drive cancerous growth; for *ROS1* this happens when rearranged or fused with other genes; for *RET* when rearranged or with small point neucleotic base substituitions; for *MET*, when there’s gene amplification or small point neucleotic base substituitions; for *ERBB2*, when amplified andoverexpressed; *B-Raf*—a gene that codifies the B-Raf protein, which regulates cellular growth, when deregulated with small point neucleotic base substituitions leads to cancerous uncontrolled cell growth; tumor suppressor genes such as *STK11*, which encodes a tumor suppressor enzyme that regulates cell growth and, when mutated with small point neucleotic base substituitions and small deletions or insertions, stops producing a normal tumor suppressing enzyme; transcription regulators like *ARID1A*—one of its functions is tumor suppression, but when mutated, it ceases this function; and finally, tumor mutational burden, which represents the number of non-inherited gene changes per million DNA bases in cancer cells, with imaging, computed tomography scan images, and digitized programmed death ligand-1 immunohistochemistry slides has been shown to improve immunotherapy prediction for non-small cell lung cancer patients [40]. Similarly, in neurology, combining functional MRI with diffusion tensor imaging enhances diagnostic accuracy in neurological disorders [41,42].

Other studies demonstrate the value of multimodal approaches in oncology: prostate cancer diagnosis benefits from integrating MRI with prostate-specific antigen biomarkers and features extracted from diffusion-weighted MRI [43], while the fusion of microscopic images, CT/MRI scans, and genomic data improves renal cancer prediction by enabling a more holistic assessment [44].

Cardiology provides further examples. Implantable cardioverter-defibrillators (ICDs) can leverage AI to generate individualized risk predictions based on demographic variables, physiological data, and arrhythmia detection [45]. The Medtronic Cardiac Resynchronization Therapy Defibrillators (CRT-D) system integrates intra-thoracic impedance, activity patterns, night heart rate, heart rate variability, and atrial fibrillation episodes to produce a heart failure risk score. The combination of physiological data from medical-grade implantable systems and AI can help predict heart-failure hospitalization in ambulatory settings and mitigate its effects [46].

In aggregate, the growing availability of heterogeneous medical data and the rapid development of AI methods provide fertile ground for multimodal models. These models enable more comprehensive analyses and yield more robust insights, ultimately supporting improved clinical decision-making.

The predictions of these models are complex and incorporate multiple data sources. The reasoning and processing behind these decisions are unknown. Explainability is key to bringing context and valid justification to the obtained outcomes.

### 3.3. Large Language Models

LLMs have undergone significant development in recent years, with several medical LLMs being introduced. Figure 2 presents the progression of these models since 2018.

Initially, most applications of LLMs in the medical domain focused on report analysis and feature extraction. Processing unstructured data, such as PDF files and scanned documents, is a time-consuming task. Traditional approaches, such as optical character recognition (OCR) and file parsing, sometimes yield inconsistent results. The application of VLLMs (Visual Large Language Models) offers a more precise alternative for these tasks. VLLMs incorporate contextual information and layout structure in an end-to-end process that allows for complex reasoning, such as summarizing a chart. Nonetheless, traditional OCR and file parsing offer more cost-effective solutions that favor scalability and provide specific coordinates for each character. A hybrid approach with OCR and file parsing, jointly with a downstream implementation of LLMs, is more scalable and cost-efficient. The decision between VLLMs and this hybrid approach presents a trade-off that must be adjusted for each situation [48].

Some studies compare humans’ and LLMs’ medical information extraction. Goel et al. [49] used Med-PaLM2 [50] to extract features from the text of the i2b2 dataset [51], comparing a human base rater, an LLM base rater, a human base rater with LLM refinement, and a human base rater with human refinement. The human base rater with human refinement solution obtained an F1-score of 91.0%, only slightly better than the LLM base rater with human refinement, which obtained an F1-score of 90.7%—a minimal disadvantage, given that the latter achieved significant time savings (26%). The F1-score represents the harmonic mean of precision and recall, where precision measures the proportion of correct positive predictions, and recall measures the proportion of correctly identified positives. The statistical difference in processing times between the approach with a human base rater and human refinement and the approach with an LLM base rater with human refinement is confirmed with a *p*-value below 0.01. The LLM’s base rater with human refinement achieved a mean time of 11.3 min, with a 95% Confidence Interval (CI) of [8.9, 13.8] min. On the other hand, the human base rater with human refinement achieved a mean time of 26.7 min, with a 95% CI of [24.2, 29.1] min. The testing set for these results consisted of 216 documents. For evaluation metrics, two prompt annotation configurations were combined. The authors designate these two annotations as: direct chunk annotation and Inside–Outside Beginning token annotations.

Further studies demonstrate that the use of models such as Llama2-7B [52] and Meditron-7B [53] to extract data from the Medical Information Mart for Intensive Care Sequential Query Language (MIMICSQL) dataset [54], which is a structured database derived from EHRs, exhibits promising results. Lovón-Melgarejo et al. [55] present two methodologies based on retrieval or extraction methods. In the former, the goal is to present a set of EHR tables given a certain natural language prompt, whereas in the latter, the goal is to generate a natural language answer for the prompt in question. As gold standard annotations, the original SQL queries from the MIMICSQL dataset were converted into natural language to create question–query pairs.

To improve the reliability of LLMs, methods such as Retrieval-Augmented Generation (RAG) emerged. RAG integrates information retrieval with text generation, where an external knowledge source is queried to provide relevant context that guides the generation process. This approach reduces “hallucination” and enhances factual accuracy by grounding the output in retrieved evidence. RAG is implemented before or during the LLMs’ clinical output generation to enhance their response. Although RAG implementation is generally beneficial, it can introduce irrelevant, outdated, or incorrect evidence and does not eliminate hallucinations [56].

Applications for LLMs include triage systems and even direct diagnostic assistance. This represents a turning point for LLMs, from background operators that served as preprocessing tools to a more pivotal role in medical decision-making. A few examples of early appearances of transformer-based methodologies in the biomedical language processing context, based on backbones such as Bidirectional Encoder Representation for Transformers (BERT) and Generative Pre-Trained Transformer (GPT), include BioLinkBERT [57], Deep Bidirectional Language-Knowledge Graph Pretraining (DRAGON) [58], BioMedLM [59], PubMedBERT [60], and BioGPT [61].

BioLinkBERT results from fine-tuning the BERT [62] architecture with a dataset of PubMed papers linked according to cross-citation. This creates a training set organized as a graph knowledge (GK) from several documents linked according to their context. DRAGON (Deep Bidirectional Language-Knowledge Graph Pretraining) introduces two parallel modules: one with a language model (LM) layer for text chunk processing, and one with a graph neural network (GNN) for GK processing. Both modules are fused bidirectionally with a GreaseLM [63] encoder. Each of these modules is fine-tuned through self-supervision: the LM module with masked tokens, the GNN module through link prediction. BioMedLM’s architecture is based on GPT-2 [64], with the addition of a custom tokenizer, Byte-Pair Encoding (BPE), that was fine-tuned on PubMed abstracts (PMAs). This tokenizer maintains key biomedical terms as a single token, which improves context. This model was fine-tuned with questions from MedMCQA [65] and MedQA [66], and was tested on the Measuring Massive Multitask Language Understanding (MMLU) [67] dataset. This process incorporates multiple-choice-specific training that increases results in standardized testing. For PubMedQA [68] and BioASQ [69], the context and question were concatenated, and the results were aligned into yes, no, and maybe.

PubMedBERT defines various tasks to fine-tune a BERT backbone, using only PubMed abstracts (PMAs). These tasks include using Whole-Word Masking (WWM), trained from scratch with only domain-related information from 30 million PMAs; Named Entity Recognition based on 1500 PMAs from NCBI-Disease [70] annotated by Crichton et al. [71], with specific relation labels for chemical and disease topics; evidence-based medicine information extraction annotated with PICO labels (Participants, Intervention, Comparator, and Outcome), with 4300, 500, and 200 PMAs for training, development, and test datasets, respectively; relation extraction, which used the Chemical Protein Interaction corpus dataset [72], which has PMAs annotated with chemical–protein interactions, the Drug–Drug Interaction corpus, annotated with drug–drug interactions [73], and the Genetic Association Database corpus with gene–disease associations [74]; a sentence similarity task with the Sentence Similarity Estimation System for the Biomedical Domain dataset [75], which has 100 pairs of PubMed sentences annotated by five expert-level annotators; a document classification task with several PMAs from the Hallmarks of Cancer (HoC) corpus [76]—each with a binary label classification using 37 fine-grained hallmarks; and lastly, question-answering tasks, using the PubMedQA dataset, with a train, development, and test split of 450, 50, and 500 questions, respectively, and multiple PMAs annotated with whether they contain an answer to a certain research question, or the BioASQ corpus dataset with a train, development, and test split of 670, 75, and 140 questions, respectively, which pairs each question with a yes or no answer and a PMA.

BioGPT uses the GPT-2_medium_ architecture as a backbone with BPE and utilizes only domain-specific data from PubMed items. It performs relation extraction from several datasets; the model uses the text as input and generates relational triplets, using the BC5CDR dataset [77] for chemical–disease relation extraction with 500 entries for training, validation, and test sets; the KD-DTI dataset [78] for drug–target interaction consisting of 12,000, 1000, and 1300 documents for the train, validation, and test sets; and the drug–drug interaction dataset DDI with a train, validation, and test split of 664, 50, and 191. Additionally, it implements a question-answering task with PubMedQA, with a data split of 450, 50, and 500 for training, validation, and testing, respectively, and a document classification task with the HoC dataset and 1580 PMA. These models were a first step in the implementation of natural language processing (NLP) in healthcare settings.

To further advance the development of NLP in healthcare, various datasets were published for uniform model benchmarking:

The MedQA dataset comprises 61,097 multiple-choice questions collected from three professional medical boards: United States Medical Licensing Examination (USMLE)—English questions, Mainland China Medical Licensing Examination (MCMLE)—simplified Chinese questions, and Taiwan Medical Licensing Examination (TWMLE)—traditional Chinese questions. The dataset is randomly split into 80% training, 10% development, and 10% testing. The training set includes 48,876 questions, which are divided into 10,178 from the USMLE, 27,400 from the MCMLE, and 11,298 from the TWMLE. The development set contains 6109 questions, composed of 1272 from the USMLE, 3425 from the MCMLE, and 1412 from the TWMLE. Finally, the testing set comprises 6112 questions, including 1273 from the USMLE, 3426 from the MCMLE, and 1413 from the TWMLE. These categories are designed to evaluate professional clinical decision-making across different linguistic and regional medical standards.

The MedMCQA dataset contains a total of 193,155 high-quality medical multiple-choice questions. To ensure model generalizability and avoid data leakage, the dataset uses exam-based split criteria rather than a random question-based split. The training set consists of 182,822 questions sourced from various mock tests and online test series created by medical professionals. The test set includes 6150 questions from real-world All India Institute of Medical Sciences entrance exams (spanning from 1991 to 2022), while the development set comprises 4183 questions from National Eligibility cum Entrance Test exams (2001 to 2022).

The PubMedQA dataset consists of 273,500 question-answering instances collected from biomedical research abstracts. This total is partitioned into three specific subsets: 1000 expert-annotated instances (PQA-L), 61,200 unlabeled instances (PQA-U), and 211,300 artificially generated instances (PQA-A). Structurally, every instance across all splits includes a question (derived from a research article title), a context (the structured abstract excluding the conclusion), and a long answer (the original conclusion of the abstract). The difference between the splits lies in their labeling: the PQA-L split contains “yes/no/maybe” labels manually provided by medical experts for testing and cross-validation; the PQA-U split contains instances that are answerable but lack labels, intended for semi-supervised learning; and the PQA-A split contains instances where questions were converted from statement titles, and labels were automatically generated based on the negation status of the original verbs.

The MMLU dataset comprises 15,908 multiple-choice questions spanning 57 tasks, divided into a few-shot development set (five questions per subject), a validation set of 1540 questions, and a test set containing 14,079 questions. Collected from freely available online sources, the dataset incorporates practice materials from the Graduate Record Examination (GRE), the USMLE, and Advanced Placement (AP) exams, organized into four broad categories: Humanities, covering qualitative disciplines like law, philosophy, and history; Social Sciences, examining human behavior through economics, sociology, and psychology; STEM, featuring calculation-heavy and abstract reasoning subjects such as physics, computer science, and mathematics; and Other, which includes specialized professional areas like business, marketing, and professional medicine. The Professional Medicine task specifically consists of highly difficult questions that require years of human study to master; while expert human test-takers in the 95th percentile achieve approximately 87% accuracy on these USMLE-based items, current models often show lopsided performance, as these questions demand expert-level problem-solving ability and the application of specialized knowledge rather than basic linguistic understanding.

The Med-PaLM and Med-PaLM2 models, developed by Google, were the first to achieve a passing score in the USMLE-style questions with an accuracy of 67.2% on the MedQA dataset, using the version from 2021 with multiple-choice options and access to relevant documentation (medical textbooks). Med-PaLM2 obtained an even higher accuracy of 86.5% [79], with the use of an ensemble refinement prompting strategy.

According to Saab et al. [80], in a project developed by Google DeepMind in 2024, the Gemini-Med model achieved 91.1% accuracy in the MedQA dataset, while ChatGPT-4 achieved a lower accuracy of 90.2%. At this time, medical-specific models were at the forefront of medical classification development.

To improve the general-purpose model’s accuracy, prompting techniques, like Medprompt, follow a series of steps to obtain better results [81]. Figure 3 schematizes these steps. The first step is a simple zero-shot method, similar to regular prompting, achieving an accuracy of 81.7%. The second step includes a random few-shot approach; this method is based on selecting random prompting examples from the training space, achieving an accuracy of 83.9%. The next step is the chain-of-thought (CoT) approach that relies on intermediate reasoning steps. The model creates sequential prompts that ensure a generated intermediate rationale behind each response. This rationale is created by the model and may contain bias, hallucinations, and even failures in logical reasoning. CoT is responsible for the largest increase in accuracy from each of these steps, achieving an accuracy of 87.3%. The fourth step begins with examples chosen from the training space with a k-nearest neighbor (kNN) algorithm to ensure they are semantically similar to each other. The process is followed by a CoT application. This step achieves an accuracy of 81.7%. In the last step, this process is repeated, with an ensemble choice shuffle, on top of the previous methods. When this study was developed, at the end of 2024, ChatGPT-4 showed a bias towards certain multiple-choice options. To tackle this issue, the choices were shuffled several times, and the most consistent answer was selected [81]. This final layer achieves an accuracy of 90.2%. The implementation of further fine-tuning led to an accuracy of 90.6%.

Medprompt increases model performance in several dimensions and benchmarks. Figure 4 shows how the ChatGPT-4 model consistently achieves better results through Medprompt.

In recent years, prompting techniques such as CoT, few-shot, and others have been inherently embedded into LLMs. In 2025, general-purpose models have consistently obtained the best results for question-answering in the MedQA dataset [82]. Recent general-purpose LLMs, such as o1, GPT-5.1, Gemini 3.1 Pro Preview, GPT-5, and GPT-5.4 (xhigh), obtained the best scores on the MedQA dataset; the top 10 for this benchmark are all general-purpose models. This exemplifies the current tendency for LLM development to be more directed towards generalized solutions.

The models were evaluated on a randomly selected subset of 2000 MedQA question–answer pairs, with the instruction to provide an answer in the format ‘Answer: [Option Letter]’. Multiple evaluation runs were conducted to assess the influence of demographic biases, and models were accessed through their respective provider APIs, including OpenAI, Google, Anthropic, and others. To minimize benchmark contamination, an “eyes-off” dataset split implementation prevented the overlap between benchmark samples and training corpora. Nevertheless, because MedQA has been publicly available since 2020, the possibility that portions of the dataset were included in the pre-training data of modern LLMs was not completely excluded [82]. Given how tightly clustered the top models are, to validate and accurately characterize the model’s performance, a 95% confidence interval (CI) was added. Table 1 shows the evolution of the scores by LLMs on the MedQA database.

The results from the best models are clearly saturated, so much so that the scoreboard has been archived by Val.ai since April 2026. Reinforcement Learning from Human Feedback (RLHF) [83] is applied to adjust LLMs towards human preferences. The most common application is with prompt and response pair demonstrations, where the most suitable is selected by a human observer. This process creates a fine-tuned assistance model from the base LLM.

RLHF can be applied in healthcare to achieve a truly personalized patient-centric medical practice. Human reinforcement creates assistance models tailored towards the patient, which can help mitigate hallucinations and build more robust, trustworthy, fair, accountable, and auditable models [84], enhancing their clinical validity.

Still, these models have weaknesses that can be exploited. One way to mitigate the bias and shortcomings of an LLM’s output is by explaining its decision. Explainability and trustworthiness go hand in hand and are necessary for the implementation of AI models.

## 4. The Importance of Trustworthiness, Transparency, and Explainability

The successful implementation of AI in healthcare depends fundamentally on trust and reliability. Given the high stakes involved in clinical decision-making, AI systems must demonstrate consistent, accurate, and robust performance. Explainability plays a critical role in fostering trust since stakeholders are more likely to rely on AI systems when their reasoning can be understood and verified. Transparent models that provide interpretable insights into their predictions enable clinicians to assess, validate, and appropriately act upon AI recommendations, while also facilitating the identification of potential biases or errors.

A clear example of these biases and errors is seen in the “Clever Hans” phenomenon. In this famous phenomenon, the model must differentiate between huskies and wolves. The model obtains reasonable results; however, its focus is not on the wolves and huskies but rather on their snowy background [85].

A process analogous to the “Clever Hans” phenomenon is described in Zech et al. [86], where the model achieves good results based on spurious correlations rather than valid image artifacts. Zech et al. [86] present a cross-hospital study for deep learning classification of pneumonia in X-ray scans. This study handled a total of 158,323 X-ray scans from three distinct institutions: National Institutes of Health Clinical Center (NIH), Mount Sinai (MSH), and Indiana University Network for Patient Care (IU). The highest internal performance within these datasets resulted from a combination of both the NIH and the MSH datasets, with an AUC of 0.931 and a 95% CI from 0.927 to 0.936. However, the model performance decreased significantly when tested on external data from IU, with an AUC of 0.815 and a 95% CI of 0.745 to 0.885. Both 95% CIs were calculated with the Clopper–Pearson method. The difference between these results was statistically significant, with a *p*-value of 0.001 according to DeLong’s test. Metadata present in the training images indicated the hospital system and department, which may have led the model to assume that metadata from a department with a higher prevalence of pneumonia in X-ray scans is a valid predictor. Instead, the model should be constrained to examine the X-ray images themselves. The researcher concluded that confounders, such as image metadata, might have biased the model. This explains the worse performance on external datasets where the metadata is non-indicative of pathology. A model tasked with differentiating between hospital systems robustly classified the MSH scans with an accuracy of 99.98% and a 95% CI of 0.9991 to 1.0000, and the NIH scans with an accuracy of 99.95% and a 95% CI of 0.9991 to 0.9997, respectively, further confirming suspicions that the model was grounding its results in confounders rather than clinically relevant data.

Explainability offers not only justification that can bring confidence to AI predictions, but it can also help rule out bad training procedures and detect a misalignment between a model’s metrics in a controlled dataset and the real world (external data).

Recent developments in LLMs are bound to shape the future of healthcare with personalized chatbots directed towards medical diagnosis. This recent development is sometimes accompanied by completely inaccurate answers. This phenomenon is referred to as “Hallucination” [87,88]. Recent advancements in models have tried to mitigate the occurrence of nonsensical responses [79]. Nonetheless, this is a problem that must be addressed.

Unfortunately, “Hallucinations” [87,88] are not the only concern related to LLMs. Ness et al. [89] explain how these models might be vulnerable to attacks. In this study, the MedFuzz algorithm was developed, which added misleading information that worked as a distractor and directed LLMs to an incorrect option. It iteratively adjusts these attacks according to the model’s confidence scores. Although these models have very good results in standardized tests, these flaws can be explored. The study reveals how the LLMs might not ground their answer in solid medical knowledge, but rather in spurious correlations.

Explainability methods may assist retrospective investigation and error detection. Beyond contributing to model performance, the implementation of XAI raises significant ethical considerations. Biases inherent in black-box models can perpetuate prejudice and injustice. Addressing these risks requires stronger alignment between ethics, legislation, and explainability to ensure safe and responsible deployment of AI in healthcare. The following chapter explores this issue in greater depth.

## 5. The Principles of Biomedical Ethics

Medical practitioners and certain groups are bound to additional ethical constraints. *The Principles of Biomedical Ethics* by Beauchamp and Childress [90] specify four principles that guide clinical and biomedical ethics. These principles are respect for autonomy, beneficence, non-maleficence, and justice.

Respect for Autonomy: Patient’s right to their freedom, decision-making, and self-assurance. The obligation to inform the patient and enable patient–provider shared decision-making with adequate understanding. Support the patient’s self-determination and independence if it does not harm others.

Beneficence: The physician’s obligation to encourage the patient’s well-being, alleviate their suffering, and promote their health.

Non-maleficence: The physician’s responsibility not to harm the patient. The obligation to act in a way that safeguards the patient’s health.

Justice: The just allocation of resources, impartial treatment of patients, and access to basic minimum healthcare.

The balance between these four principles must be present in both clinical investigation and practice. Explainability can bring a new dimension to upholding each of these principles.

The increased transparency in an otherwise opaque black box is part of the factors responsible for the increased autonomy in patient–provider shared decision-making. A justified model answer provides a stronger basis for an informed decision by all parties.

This transparency is responsible for robust, concise, and justifiable medical decisions that are of key benefit to upholding both the beneficence and non-maleficence principles.

To uphold the justice principle, the reach of quality healthcare must expand to all those in need. A reliable AI model with proper justification for its answers presents more equitable access to healthcare, for example, in impoverished areas where access to medical experts is limited. Despite accessibility barriers to digital infrastructure and the presence of biased datasets towards specific groups being factors that introduce inequity into an AI-dominated healthcare framework, overall, more people can experience the benefits of quality medical care.

The World Health Organization (WHO) stands by these principles and provides directives that abide by the core of biomedical ethics. A set of norms for ethics and governance is defined in [91]. These norms align with the direction that should be followed in this regard. The necessity for these norms indicates why guidelines and legislation are paramount in this field.

## 6. Current Legislation in Place

For a medical device with AI implementation to be compliant with the CE Mark, it must uphold Article 48 of the EU AI Act, which provides a link between the CE Mark and the EU AI Act. According to Article 48, the products classified under high-risk AI systems by Article 6 must follow Article 8, which mandates compliance with Articles 9 through 15. Article 40 enforces standardization and legal requirements with existing European and international standards. This presents a final milestone that grants the official seal of approval for obtaining the mark, as per Article 48 [3]. Figure 5 presents a diagram of the legislative procedures in the EU AI Act.

As of August 2026, the implementation timeline of the EU AI Act has been modified by the Digital Omnibus on AI (Regulation (EU) 2026/1744) [92], effective the 27th of July 2026. The amendment postpones the application of several obligations relating to high-risk AI systems to allow additional time for the development of harmonized standards and implementation mechanisms. Under the revised timetable, requirements for standalone high-risk AI systems (Annex III) are enforced as of the 2nd of December 2027, while obligations for high-risk AI systems embedded in products covered by sector-specific legislation (Annex I) are mandatory starting the 2nd of August 2028.

High-risk AI systems must be registered in the EU database established under the AI Act by their providers or, where applicable, their authorized representatives. Initially, the enforcement date for registering high-risk AI systems was the 2nd of August 2026; however, during the development of this study, the database was not yet operational. It is expected to be operational as of late 2027 and provide public access to information on most high-risk AI systems, except for law enforcement and other sensitive uses. The requirements for standalone high-risk AI systems (Annex III) were postponed to the 2nd of December 2027; however, this addendum does not explicitly state that the registration for such systems is also postponed. Nonetheless, the absence of a functional database makes the registration unfeasible.

Furthermore, the processing of patient information must comply with the GDPR, which establishes requirements for the lawful processing of personal and health data, including data minimization, purpose limitation, transparency, security, and data subject rights. Consequently, healthcare AI systems must address not only cybersecurity and AI governance requirements but also data protection obligations throughout the lifecycle of the system. Articles 9–15 are the core Articles responsible for requirements pertaining to products under high-risk AI systems and are outlined as follows:

• Article 9—Risk Management System: Maintain a continuous risk-management system throughout the documentation of the system’s lifecycle. Consider the system’s impact on vulnerable groups and underage individuals.
• Article 10—Data and Data Governance: High-risk AI systems must use training, validation, and testing data that mitigate potential biases and are relevant and representative.
• Article 11—Technical Documentation: Providers must prepare and keep up-to-date technical documentation that demonstrates compliance with the EU AI Act’s requirements.
• Article 12—Record-keeping: Requires automatic event-logging capabilities over the entire system’s lifecycle and ensures traceability in record-keeping.
• Article 13—Transparency and Provision of Information to Deployers: Ensures that models are developed with sufficient transparency so that deployers can interpret the system’s output and use it appropriately.
• Article 14—Human Oversight: The system should allow for human oversight and avoid over-reliance, focusing on output explainability.
• Article 15—Accuracy, Robustness, and Cybersecurity: Focuses on technical aspects to ensure robustness and accuracy. Both of these points relate to the benefits of explainability applications.

Explainable models play an important role in further aligningmodel requirements within the available legislation. These methods provide justification that leads to more transparent and robust options. This increased transparency correlates with what is expected in the Articles above. The EU AI Act enforces transparency through Article 13, while Article 14 enforces human oversight. The combination of these two articles gives greater insight into the decision-making process that supports respect for patients’ autonomy, which is stated in the biomedical ethics principles.

Higher transparency and performance accuracy translate into the principles of beneficence and justice, which are key biomedical ethics principles. This illustrates how the EU AI Act is constructed to ensure proper healthcare for patients and safer decision-making for physicians.

In addition to the EU AI Act, other concurrent legislation must be complied with—legislation regarding cybersecurity, data protection, and even copyright.

In terms of cybersecurity, legislation such as the NIS2, more specifically Directive (EU) 2022/2555 [93], enforces stricter risk management, mandates incident reporting within 24 h, and enforces cybersecurity compliance from malware checking in open-source software dependencies to cloud service risks. Moreover, legislation such as the Cyber Resilience Act, specifically Regulation (EU) 2024/2847 [94], mandates that hardware and software components have enforced security regulations throughout their lifecycle. Lastly, the Cybersecurity Solidarity Act enforces cooperation between states to create a cyber shield that protects organizations and citizens from massive cybersecurity threats with early detection, relying on cross-border monitoring and a trusted network of specialists tailored towards this situation. Figure 6 represents the mechanism behind this cybersecurity layout.

As for copyright, the EU has mandatory legislation as well, under Directive (EU) 2019/790 [95]. This directive seeks to harmonize protection across member states. It focuses on protecting producers and artists. AI developers must verify data mining and training against copyrighted materials to avoid copyright infringement. This directive offers one more layer of protection against data misuse and abuse, and against unethical and unlawful data practices.

All these practices converge on one important point: the safeguarding and protection of the patient’s health data. Cybersecurity measures are crucial to ensure that there are no data leaks. These measures enforce privacy and security, crucial to abiding by the non-maleficence principle of biomedical ethics.

## 7. Potential Contributions of XAI to Ethical and Regulatory Requirements

Ensuring adherence to ethical standards and legal frameworks requires the fulfillment of specific technological requirements. In this regard, just as techniques such as Federated Learning [96] have been adopted to strengthen the security of training applications, explainability methods can similarly contribute to safeguarding ethical principles and supporting regulatory compliance by enhancing transparency and accountability in AI systems.

Explainability methods can enhance transparency and provide information to deployers. These methods contribute in part to the enforcement of Article 13 of the EU AI Act, which obliges providers of high-risk AI systems to guarantee transparency and provide clear instructions for use. A justification for the models’ outcomes is the best way to determine if the outputs are unfairly discriminatory.

Although Article 13 of the EU AI Act does not mandate specific explainability techniques, methods such as saliency maps, attention maps, SHAP values, and other XAI approaches may support compliance by helping deployers interpret model outputs and understand system limitations. These techniques can complement the transparency and information requirements established by Article 13, particularly in healthcare settings where the interpretation of AI outputs is critical for clinical decision-making and human oversight.

This increased transparency is also responsible for enhancing the autonomy of the user. The provision of reliable explanations can improve the user’s understanding of the problem and consequently increase the assurance in their decision.

The use of explainability is key to the development of reliable, trusted legal and ethical models. In the next chapter, there is a more in-depth description of technical explainability approaches. A solid understanding of these methods can bring insight into how to apply them efficiently.

## 8. Exploratory Data Analysis and Technical Approach to Explainability

There are a variety of approaches to explainability methods and their development. From classical statistics and data-derived explanations that are completely independent of the models in question, to explainability methods that are intrinsic to the model itself. Figure 7 shows an extensive taxonomy diagram of these models, providing an overall view of what methods of explainability exist and how to apply them.

In this section, there is an overview of four principal areas associated with transparency in deep learning deployment: exploratory data analysis, white models, model-agnostic methods, and model-based methods.

### 8.1. Exploratory Data Analysis

Data-derived explanation methods facilitate classification tasks by leveraging simple data transformation techniques. Their goal is to manipulate and simplify complex data so that it is more understandable. This process enhances and eases the classification task. There are three distinct data-derived approaches: classical statistical methods, dimensionality reduction, and clustering.

#### 8.1.1. Classical Statistical Methods

Classical statistical methods, including Analysis of Variance (ANOVA) tables and probability distribution functions, provide a foundation for comprehensive analysis. They support inference-based approaches such as t-tests and ordinary least squares (OLS) regression to deliver interpretable measures of feature importance. By employing a variety of continuous and discrete probability distribution functions, real-world problems can be modeled effectively, allowing simple, data-driven decisions to be made directly from the observed values [97].

In medical practice, the use of *T*-scores and *Z*-scores for bone mineral density (BMD) assessment is an example of probability distribution functions’ medical domain applications. These measures compare a patient’s values against a Gaussian reference distribution, employing the mean and standard deviation of the reference population, as expressed by the following equations [98]:

(1) $$T-score=\frac{x-\mu_{1}}{\sigma_{1}}$$

(2) $$Z-score=\frac{x-\mu_{2}}{\sigma_{2}}$$

The *T*-score compares an individual’s BMD with that of a same-sex healthy young adult reference group, characterized by a mean of $\mu_{1}$ and a standard deviation of $\sigma_{1}$. The Z-score reflects a comparison with a population of similar age and sex, defined by a mean of $\;\mu_{2}$ and a standard deviation of $\sigma_{2}$ [98]. Both measures are widely employed in the diagnosis and clinical management of osteoporosis [99].

#### 8.1.2. Dimensionality Reduction

Data dimensionality reduction encompasses methods such as principal component analysis (PCA), linear discriminant analysis (LDA), and independent component analysis (ICA), among others. These approaches generate alternative representations of the data space, which can uncover latent patterns and yield meaningful insights. Dimensionality reduction can compromise semantic interpretability because the resulting components represent combinations of multiple original variables, making their individual contributions and clinical meaning difficult to interpret.

PCA projects data onto its principal components, capturing the directions of maximum variance along orthogonal axes. This transformation reduces the data to a lower-dimensional space. The resulting visual representation of these components, as shown in Figure 8, facilitates the interpretation of potential correlations [100].

This method demonstrates broad applicability in healthcare, ranging from the evaluation of clinical and biochemical parameters in children with congenital adrenal hyperplasia [101] to neurological contexts such as electroencephalography analysis for epilepsy detection [102] and the development of brain–computer interfaces [103].

LDA separates the data with a linear threshold to maximize the distance between classes and minimize the variance in each class, as presented in Figure 9 [104]. This method has applications in early disease detection from EHRs with multiple input variables, where dimensionality reduction facilitates the interpretation and exploration of complex, multivariable datasets [105].

ICA achieves dimensionality reduction through latent component identification based on maximizing statistical independence of underlying sources, generally with non-Gaussianity assumptions, as illustrated in Figure 10 [106,107]. In healthcare, it has been applied to magnetoencephalography for signal-to-noise enhancement, enabling the separation of signal components and the extraction of neural activity with greater precision [108].

Variants of the previous methods include Fisher’s Wishart Discriminant Analysis for EHR-based early disease detection [105], Kernel PCA for optimizing lung cancer prediction [109], and Sparse PCA for advancing cancer research [110]. These methods illustrate the diversity of approaches for modeling and extracting meaningful features from complex biomedical data.

Collectively, component analysis techniques not only facilitate the derivation of actionable insights from high-dimensional datasets but also enhance interpretability and data visualization, thereby supporting more informed and effective clinical decision-making.

#### 8.1.3. Clustering Methods

Simple clustering algorithms, such as K-means, hierarchical clustering, Gaussian Mixture Models (GMMs), and density-based spatial clustering of applications with noise (DBSCAN), offer valuable tools for exploratory data analysis. Key limitations include sensitivity to feature scaling and the choice of distance or similarity metric, as well as the potential for resulting clusters to lack clear clinical interpretability.

K-means is an iterative, centroid-based method that partitions a dataset according to distance metrics and has been applied in healthcare for pattern analysis, notably through a variant called G-means [111], which demonstrates how even simple or slightly modified algorithms can impact clinical applications.

Hierarchical clustering has both an agglomerative and a divisive approach. Agglomerative clustering follows a bottom–up process, starting with each point as a separate cluster and merging them iteratively based on inter-point distances until a single cluster remains. Conversely, divisive clustering employs a top–down strategy, beginning with all points in one cluster and recursively splitting them until each point forms its own cluster. Yang et al. [112] applied K-means, hierarchical clustering, and their modifications to develop smart solutions integrating structured and unstructured data for patient risk monitoring.

GMMs are probabilistic clustering models that represent data as a combination of multiple normal distributions, each corresponding to a cluster. Data points can belong to multiple clusters, with associated probabilities reflecting the degree of membership. In healthcare, GMMs have been applied to track eye movements for the diagnosis of Nystagmus [113].

DBSCAN classifies points as core, border, and noise, aggregating density-connected points defined by a user-specified radius (eps), as illustrated in Figure 11 [114]. This method is applicable for data mining and exploratory analysis of complex datasets in healthcare [115].

### 8.2. White Models

From machine learning to deep learning, increasing model complexity often creates a trade-off between performance and interpretability. More complex models are opaque, whereas simpler methods, such as decision trees, OLS regression, and rule-based approaches, offer intrinsic, ante hoc interpretability. The future of XAI aims to balance high model performance with interpretability, addressing the traditional trade-off between model complexity and explainability, which is shown in Figure 12. The complexity of an algorithm should be the minimum possible relative to a specific task, to favor both performance in clinical utility and interpretable decision-making. Ding et al. [116] explore this trade-off and conclude that a minimal complexity should be established for each task.

Decision trees, used for both classification and regression, process categorical inputs through a series of sequential binary decisions. Ensembles of trees form random forests, which have demonstrated promising results in healthcare applications [117,118]. It is worth noting that although shallow single decision trees are highly interpretable, the development into complex random forests with various trees and multiple nodes is far more difficult to explain. Generalized linear models (GLMs) employ linear predictors with coefficients for independent variables, fine-tuned iteratively to maximize likelihood estimation according to selected probability distribution functions’ [119,120]. Generalized Additive Models (GAMs) extend GLMs by combining parametric models with non-parametric functions, such as B-splines or kernel smoothers, to create a single, interpretable model [121]. Waqas et al. [122] demonstrated how GAMs can be applied for ischemic and hemorrhagic stroke monitoring in cardiology patients for over a year, achieving good results in healthcare data processing and analyses.

Bayesian models leverage conditional probabilities. Equation (3) represents the conditional probability equation, where A and B are two dependent variables. Bayes’ Theorem derives from Equation (3) and provides a relationship between conditional probabilities that can be used to update the probability of a hypothesis based on observed evidence [123]. Directed acyclic graphs (DAGs), schematized in Figure 13, organize the dependencies between variable nodes (a, b, c, d, and e) [124]. DAGs provide a general graphical representation of variables and their directed relationships without necessarily implying causality. Bayesian networks extend DAGs by associating probability distributions with the graph to represent conditional dependencies and support probabilistic inference. Causal DAGs, in contrast, assign a causal interpretation to the directed edges and therefore require explicit assumptions or substantive knowledge about the underlying data-generating process. These models have applications in cardiac, cancer, psychological, and lung disorders, although their practical deployment in healthcare remains limited [125,126,127].

(3) $$P\left(B|A\right)=\frac{P(A\cap B)}{P(A)}$$

Knowledge graphs share the concept of nodes and edges with DAGs, with specific knowledge information added. They encode knowledge in a way that allows for reasoning and inference. The nodes represent entities, and the edges represent relationships between entities that provide semantic information and knowledge. These graphs are used for neuro-symbolic approaches and can be combined with deep learning methodologies. This topic is further developed in Section 8.4.3 Neuro-Symbolic-Based Methods.

Support Vector Machines (SVMs) define hyperplanes iteratively to separate high-dimensional data and select the hyperplane that best separates each class. Although linear SVMs are highly interpretable, non-linear SVMs are not intrinsically transparent. SVMs have been used in prognosis predictions for laryngeal, prostate, hepatic, and renal cancers [128,129,130,131].

Ensemble models combine multiple methods, increasing complexity and reducing interpretability. Techniques include bagging, boosting, stacking, and voting. Bagging trains models on different data partitions and aggregates predictions; boosting sequentially trains models to correct prior errors; stacking uses a meta-classifier to combine predictions; and voting merges model outputs via hard or soft votes. Ensemble methods have been applied to predict outcomes for heart disease, kidney disease, liver disease, skin disease, and diabetes [132].

Deep learning models, while achieving high predictive performance, are opaque and lack direct interpretability, highlighting the need for monitoring tools such as model logs to detect failures and ensure trustworthiness. Nonetheless, simpler models can achieve comparable results, emphasizing the importance of tailoring the method to the specific problem while ensuring transparency, safety, and confidence in healthcare applications [133].

### 8.3. Model Agnostic Methods

Post hoc methodologies play a pivotal role in evaluating model explainability, offering approaches that can be applied across diverse domains. Among these, model-agnostic methods are particularly notable, as they operate independently of the underlying model, giving them a universal aspect. Representative techniques within this category include Local Interpretable Model-Agnostic Explanations (LIME), SHapley Additive exPlanations (SHAP), Shapley, and Randomized Input Sampling for Explanation (RISE) of black-box models.

The LIME methodology approximates the behavior of complex classifiers by testing model performance with and without specific input features. For image data, these features are typically "superpixels"—clusters of adjacent pixels forming meaningful patches. In textual applications, they correspond to the presence or absence of word embeddings. The objective is to generate an interpretable local model that mimics the original classifier. The match between these localized approximations and the general classifier enables the identification of features that exert the greatest influence on the model’s prediction [134].

A frequently cited illustration of LIME’s utility is the "husky versus wolf" classification task, presented in Section 4. In this case, LIME demonstrated that the classifier relied heavily on background features, such as snow, rather than the animals themselves, to justify its predictions [134].

Applications of LIME in healthcare contexts are proven examples of its relevance. For instance, studies have shown that LIME can identify features such as glucose level, body mass index, and age as being most influential in heart disease prediction models [135]. LIME image applications in X-rays delimit regions responsible for the model’s decisions. Figure 14 illustrates the application of the LIME method for explainability in viral pneumonia detection [136].

The Shapley values are grounded in cooperative game theory. It evaluates the contribution of individual features by comparing model predictions when a feature is present against predictions when that feature is withheld. The value function and coalition expectation represent the core mathematical framework behind the expectation of outcome generated by a coalition of feature values. The expectation of outcome is determined when a specific coalition of features is known. To calculate this expectation for subsets where certain features are absent, the method utilizes a reference distribution (or background distribution) to sample values for the absent variables, effectively estimating them. Reference distribution algorithms assume that individual features are distributed independently, which simplifies the process of sampling from the reference distribution but impacts how correlations are handled during the calculation of marginal contributions. Ultimately, the SHAP value is derived by averaging these marginal contributions—the differences between expectations with and without a feature—across all possible feature permutations to ensure a fair and consistent allocation of the model’s prediction [137,138]. Formally, Shapley values are given by Equation (4) [138]:

(4) $$\phi_{i}=\underset{s\subseteq F\backslash \{i\}}{\sum }\frac{\left|S\right|!\left(\left|F\right|-\left|S\right|-1\right)!}{\left|F\right|!}[f_{S⋃\left\{i\right\}}\left(x_{S⋃\left\{i\right\}}\right)-f_{S}(x_{s})]$$

Here, F denotes the full feature set, and S is a subset of features excluding i. $S\cup \left\{\mathrm{i}\right\}\;$ represents the S subset including the feature i. The i variable represents the features analyzed for the given Shapley values, and f denotes the surrogate model used to compute Shapley values. The fraction $\frac{\left|S\right|!\left(\left|F\right|-\left|S\right|-1\right)!}{\left|F\right|!}$ is the standard weighting used in game theory to ensure fair distribution, accounting for all possible permutations in which features can be added to the coalition. The $\left[f_{S⋃\left\{i\right\}}\left(x_{S⋃\left\{i\right\}}\right)-f_{S}(x_{s})\right]$ represents the marginal contribution of feature *i*. The subset where the summation is defined is *S*⊆*F*∖{*i*}, to include all possible subsets that do not already include feature *i*. In essence, the SHAP value provides a normalized difference between the surrogate model’s prediction with and without the feature i.

The SHAP framework builds on Shapley value regression by incorporating aspects of LIME, subject to three essential properties:

1. Local accuracy: Predictions from the surrogate linear model must match those of the original model for the given instance.
2. Missingness: When a feature is absent from the surrogate model, its contribution to the output must be null.
3. Consistency: If the model’s dependence on a given feature increases, the corresponding SHAP value must not decrease, as represented in Equations (5) and (6) [137].

(5) $$f_{x}^{\prime }\left(z^{\prime }\right)-f_{x}^{\prime }\left(z^{\prime }\backslash i\right)\geq f_{x}\left(z^{\prime }\right)-f_{x}(z^{\prime }\backslash i)$$

(6) $$\phi_{i}(f^{\prime },\;x)\geq \phi_{i}(f,\;x)$$

In these formulations, f denotes the surrogate model used to compute SHAPley values. The $f^{\prime }$ represents the surrogate model when it is more reliant on feature ⅈ, z′ represents the input variable, including the feature i, while z\ⅈ corresponds to the input with i=0. $\phi_{i}$ quantifies the Shapley value for the feature i under evaluation [137].

The SHAPley approach has demonstrated its effectiveness in various domains, including healthcare. For example, it has been successfully employed to identify features of critical importance in the prediction of cerebral infarction [139].

The RISE method [140] is occlusion-based; it uses several randomly generated binary masks to hide portions of the input image. Each time an input image passes through the neural network, the resulting prediction score is attributed to the exposed parts of the input, creating a heatmap. The weighted sum of all the heatmaps combined leads to the RISE prediction.

RISE has been applied to three-dimensional computed tomography (3DCT) scans for brain hemorrhage detection [141]. The axial slices demonstrate that RISE predictions accurately highlight the regions corresponding to the hemorrhage, providing clinically interpretable visualizations that can assist radiologists in identifying affected regions, as illustrated in Figure 15.

LIME and SHAP are broad methods that encompass tabular and image modalities. Although it is not included in this section, variations in SHAP can be applied to images, as is the case for Deep SHAP, which is mentioned in Section 8.4.2 Heatmap Methods.

Nazat et al. [142] analyze the consistency of the SHAP and LIME methods using two stability metrics (global stability and local stability), which quantify the intersection size of top-ranked features identified across multiple runs under identical settings. Global stability assesses this consistency across a large batch of samples to ensure overall model reliability, whereas local stability focuses on maintaining consistent explanations for a single instance. The stochastic nature of these processes from initial random sampling leads to different outputs. The experimental results across both the VeReMi [143] and Sensor [144] datasets, jointly with multiple models: decision tree, random forest, deep neural networks, kNN, and SVMs, consistently demonstrate that SHAP outperforms LIME in both metrics. Due to its superior performance in providing reproducible and consistent insights, the researchers identify SHAP as a more trustworthy tool for safety drivers and security analysts monitoring autonomous driving systems.

### 8.4. Model-Based Methods

Neural networks are often regarded as opaque black boxes, in which achieving interpretability remains a considerable challenge. To mitigate this limitation, several post hoc explainability techniques have been developed. These methods can generally be categorized into gradient-based approaches, activation-based approaches, and hybrid approaches that combine both.

In this section, there is an overview of saliency maps and their derivatives. An approach to heatmap-related methods, and in the last section, there is a point regarding LLMs’ explainability.

#### 8.4.1. Saliency Maps

Gradient-based methods rely on the derivative computations obtained during the backpropagation process of neural network training. Since the input size of the network corresponds to the dimensions of the image data, the derivatives can be mapped pixel by pixel. The resulting values reflect the sensitivity of the output to changes in each input pixel.

This pixel-wise attribution produces saliency maps, which visualize the influence of input regions on the prediction. For RGB images, the maximum gradient value across the three channels is typically selected. Saliency maps can also be thresholded to highlight only the most relevant regions of the image [145].

This method can be applied to medical images. In Keles et al. [146], saliency maps were used to interpret the focus of an MRI tumor classification model. The maps displayed the contours of the tumor in correctly classified images, while ignoring them in erroneous predictions, as illustrated in Figure 16.

Although saliency maps are useful, there is a tendency for them to focus on edges irrelevant to the model’s classification. Rectified Gradients (RectGrads) mitigate this issue. They attribute an importance score for determined units by multiplying the activation and the propagated gradient of their neurons. They only propagate the gradient when the unit’s importance score is over a certain threshold to achieve class-related explanations [147].

In addition to using only gradients, convolutional neural networks (CNNs) themselves can provide meaningful saliency maps. Originally proposed for unsupervised learning [148], DeconvNet has been repurposed for explainability tasks by Zeiler et al. [149]. Specific layers from a classification model can be passed through a DeconvNet to reveal patterns responsible for the prediction. Figure 17 illustrates how DeconvNet can be applied to medical images.

In addition to DeconvNet adding detail to explanations [145,149], Springenberg et al. [148] demonstrated improvements by combining standard backpropagation with DeconvNet. This hybrid approach, termed guided backpropagation, produces explanations with enhanced sharpness and clarity.

Healthcare applications using Guided Backprop and RectGrads are further explored in the Heatmap Methods section below. These methods can provide meaningful explanations in image analysis, aiding physicians’ medical decision-making process with the required trustworthiness and reliability.

Other refinements to saliency maps include SmoothGrad [151], which produces sharper and more robust explanation maps. It generates multiple noisy copies of the input image, computes saliency maps for each, and averages the results. This method improves precision by incorporating Gaussian noise with a user-defined standard deviation and number of samples.

Singh et al. [150] trained neural networks with OCT images from the University of California, San Diego (UCSD) OCT dataset [152] to detect ophthalmic pathologies. To obtain an explanation for each of the model’s decisions, several techniques were applied. Figure 17 illustrates how the DeconvNet and SmoothGrad explainability methods perform in this study.

SmoothGrad can also be combined with Guided Backprop [151], producing explanations that benefit from both methods: the detail of Guided Backprop and the stability of SmoothGrad.

#### 8.4.2. Heatmap Methods

Feature maps in CNNs represent a straightforward heatmap that is indicative of a model’s inner workings. The activations represented are not automatically faithful or clinically interpretable. However, they contain valuable information about the model’s decision-making process. The activation values in these maps indicate the specific image regions that the model focuses on [149].

Azam et al. [153] analyzed sequential feature maps to gain insight and further leveraged an interpretable approach towards skin cancer image analysis. After extracting 17 geometric features and 6 intensity-based features from each feature map, patterns between feature maps of each class revealed part of the CNN’s inner working mechanisms, as illustrated in Figure 18, where the various feature maps from multiple layers associated with an image classification process are represented. Each image represents the feature map associated with a single layer.

Another useful example of explainable heatmaps is the Deep Learning Important FeaTures (DeepLIFT) method, a backpropagation-based method that assigns contribution scores to inputs in a single backward pass [154]. Unlike standard gradient methods, DeepLIFT is reference-based: it compares the activation of each neuron t to that of a predefined reference $t^{0}$, resulting in $\Delta t=t-t^{0}$. The same process is applied to the neuron’s inputs, obtaining $\Delta x_{i}$. This reference-based method has meaningful attributions even when gradients are zero or discontinuous [154]. It is particularly useful with Rectified Linear Unit activation functions, given their non-differentiability at point 0, formulated by max(0, input) [155].

A contribution score is attributed to a set of input neurons’ reference difference $\Delta x_{i}$ for an intermediate layer of the model, in relation to its activation reference distance Δt. This score is denoted $C_{{∆x}_{i}∆t}$, in relation to input $\Delta x_{i}$ and the output Δt. This framework ensures consistent activation attribution across layers.

DeepLIFT can be integrated to form Deep SHAP, a combination of SHAP and DeepLIFT in a hybrid explainability method. This method combines the layer-wise decomposition of DeepLIFT with the consistency guarantees of SHAP [137]. The layer-wise properties of DeepLIFT facilitate the construction of intuitive heatmaps, while SHAP ensures stable and theoretically consistent feature attributions. This integrated approach has been applied in healthcare by van der Velden et al. [17] to enhance the explicability of volumetric breast density estimation models using MRI images. Their study demonstrated how Deep SHAP highlights the anatomical structures the model relies upon for density estimation, thereby offering physicians valuable insights to either trust or question a model’s decision. Although matching with correct anatomical signs is a good indicator of validity within the model’s prediction, it does not necessarily establish explanation fidelity, calibration, or clinical validity. As shown in Figure 19, correctly classified cases emphasize glandular and fatty tissue (Figure 19a), whereas misclassified cases incorrectly attribute relevance to the pectoral muscle (Figure 19b).

Class Activation Mapping (CAM) [156] is an activation-based technique that generates class-specific heatmaps. CAM introduces a Global Average Pooling (GAP) layer before the Softmax classification layer. The weights from the GAP layer are multiplied by the feature maps of the final convolutional layer, producing a weighted sum that highlights the most discriminative regions for a given class. The process is illustrated in Figure 20.

CAM has several healthcare applications; for example, Sampaio et al. [157] use CAM to detect lesions in mammograph images. This method highlights the lesions in the mammograph as a heatmap. Figure 21 illustrates the highlighted lesions in question.

Gradient-weighted Class Activation Mapping (Grad-CAM) [158] generalizes CAM, making it applicable to a wider range of architectures. Unlike CAM, which requires a GAP layer, Grad-CAM leverages gradient information from the last convolutional layer to weight convolutional feature maps and then upsample a coarse localization map. This results in class-sensitive heatmaps that not only highlight discriminative regions but can even capture counterfactual explanations that may point to alternative class predictions with only a slight modification—the negation of the gradients from a predicted class.

The Grad-CAM methodology has been applied in healthcare to highlight important regions for the model’s prediction. For instance, Mascarenhas et al. [159] employed Grad-CAM to enhance the explainability of anastomosis prediction in colorectal surgery, thereby visualizing the image regions most relevant to the model’s decision-making. As illustrated in Figure 22, this approach highlights salient areas, thereby providing greater transparency and supporting a qualitative inspection of the model’s predictions.

Liu et al. [160] take CAM a step further, with the introduction of a novel method, Class Activation Map Guided Back Propagation (CGBP). Similarly to Grad-CAM, this method also generalizes the architecture applicability of the underlying model to models without a GAP layer. This method compares a target and a hostile saliency map; values in the target map that are lower than those present in the hostile map are erased. These maps begin with CAM; the attributions for the target map are directed to the target class, while the rest is mapped from the opposite class. The CAM map is then thresholded, multiplied by the last layer’s gradients, and RectGrad is applied. This method was compared with the regular RectGrad and Guided Backprop, as exemplified in Figure 23.

The Layer-wise Relevance Propagation (LRP) method [161,162] redistributes the relevance of the output neuron backward through the network. The relevance associated with a determined neuron $R_{i}^{\left(\;l\right)}$ is defined as the sum of the relevance from all incoming neurons ${\sum R}_{i\leftarrow k}^{\left(\;l,\;l+1\right)}$. The organization of the relevance associated with each layer $R_{i}^{\left(\;l\right)}$ and between layers $R_{i\leftarrow k}^{\left(\;l,\;l+1\right)}$ can be interpreted, as shown in Figure 24.

In $R_{i}^{\left(\;l\right)}$, the i represents the number of the neuron in question, and the l represents the number of the layer. In $R_{i\leftarrow k}^{\left(\;l,\;l+1\right)}$, the l continues to represent the layer, while the i represents the following neuron, and the k represents the previous neuron.

The relevance assigned to each neuron depends on both its activation and weight, as defined in Equation (7) [161,162].

(7) $$R_{i\leftarrow k}^{\left(l,\;l+1\right)}=R_{k}^{\left(\;l+1\right)}\frac{a_{i}w_{ik}}{\sum_{h}a_{h\;}w_{hk}}$$

Here, $a_{i}$ is the activation in the neuron i, and $w_{ik}$ is the weight connecting neurons i and k. The h indicates the sum of all the neurons from the previous layer. The authors present two methods for numerical stability and to smooth or tune the resulting heatmap. The ε-rule is a redistribution formula that utilizes a small “stabilizer” term ε to prevent numerical instability when weighted neuron activations are near zero, as represented in Equation (8). The ε term is multiplied by a sign function that returns 1 for positive inputs, −1 for negative inputs, and 0 for a null input. While this rule is effective at smoothing out noise, larger ε values cause relevance to dissipate during backpropagation, which leads to incomplete or sparse explanations.

(8) $$R_{i\leftarrow k}^{\left(l,\;l+1\right)}=R_{k}^{\left(\;l+1\right)}\frac{a_{i}w_{ik}}{\sum_{h}a_{h\;}w_{hk}+\varepsilon *sign(\sum_{h}a_{h\;}w_{hk})}$$

The αβ-rule presents the α and β parameters used in a conservative redistribution rule—where α scales positive evidence and β mitigates the negative evidence supporting a class. The authors suggest the use of a β≤0 and to maintain α=1−β. This method is not designed to absorb relevance, but rather to augment the significance of positive evidence and mitigate the significance of negative evidence for the model’s prediction. A higher modular β value translates into less visual sparsity and a more focused heatmap, as it more aggressively subtracts contradictory evidence to highlight specific, class-relevant structural features such as contours or object parts [161,162].

LRP has emerged as a valuable technique for enhancing explainability in healthcare applications. Bohle et al. [163] applied LRP to the classification of AD using MRI data, analyzing the contribution of inputs both at the voxel level and the feature level to the network’s predictions. As illustrated in Table 2, the voxel-wise visualization highlights brain regions identified by LRP as relevant to the classification outcome. Notably, these regions align with the established domain knowledge, underscoring LRP’s potential as a reliable tool for improving transparency and explainability in computer-assisted medical decisions. There are 193 AD patients with 969 scans. It is worth mentioning that, although anatomical agreement between the LRP heatmap and domain knowledge points towards the plausibility of the prediction, it alone does not suffice as an irreproachable metric for reliability; factors such as reproducibility and sensitivity to model randomization are key factors to be considered.

Gradient and activation-based approaches provide complementary insights into the inner workings of neural networks. Gradient-based methods such as saliency maps, SmoothGrad, guided backpropagation, and DeepLIFT emphasize pixel-level contributions, while activation-based methods like feature maps, CAM, and LRP highlight higher-level regions of importance. Hybrid approaches that combine gradients and activations, such as Grad-CAM or Guided Backprop + SmoothGrad, have proven particularly effective in enhancing explainability while maintaining fidelity to the original model.

#### 8.4.3. Neuro-Symbolic-Based Methods

Healthcare approaches include multiple logic-based systems that comprise structured guidelines and protocols to examine healthcare and medical-related fields. These systems are used to encode EHRs. They comprise hierarchical dictionaries that present concepts related to healthcare and allow for logical processing.

Systems such as Logical Observation Identifiers Names and Codes (LOINC), Systematized Nomenclature of Medicine—Clinical Terms (SNOMED CT), and RxNorm provide structured frameworks that organize medical-related concepts. All these systems are part of the Unified Medical Language System (UMLS).

LOINC, initiated in 1994 by the Regenstrief Institute, is an international clinical terminology designed to provide universal identifiers for health measurements, laboratory observations, and clinical documents. It utilizes a six-dimensional semantic data model to precisely define each unique observation based on its component, property, time aspect, system, scale, and method. LOINC’s primary function is to standardize the “question” in health data exchange, identifying exactly what is being measured to allow data from idiosyncratic local systems to be accurately aggregated and compared [164].

SNOMED CT (Systematized Nomenclature of Medicine Clinical Terms) is the world’s largest and most comprehensive clinical reference terminology, featuring a context-rich, multi-hierarchical structure to represent concepts such as clinical findings, procedures, and body structures. It is built upon a formal description logic framework (specifically the ℰℒ++ language—a lightweight description logic that allows fast reasoning for classification and instance checking), which enables the unique representation of clinical meaning through defined relationships between concepts. While covering a vast range of medical specialties, it is frequently employed to provide the “answer” or value to a clinical observation, such as a specific diagnosis or a qualitative laboratory result [165].

RxNorm is a standardized drug terminology developed by the National Library of Medicine to integrate various drug knowledge bases and address the lack of uniformity in medication naming. It creates a standardized nomenclature for every drug entity, uniquely identifying medications based on a definitional triad of ingredient, strength, and dose form. RxNorm serves as the mandatory nomenclature for electronic prescribing and medication reconciliation in the U.S., providing permanent concept identifiers that facilitate the safe and interoperable exchange of medication data across disparate systems [166,167].

The Unified Code for Units of Measure (UCUM) is a code system designed for the unambiguous electronic communication of units of measure across international science and healthcare. Its role is to eliminate clinical interpretation errors caused by inconsistent abbreviations, typographical details, or different local conventions for expressing units. By standardizing units into a single machine-readable format (e.g., ensuring “μg/day” is not confused with “mcg/24 h”), UCUM ensures that result values are handled consistently by clinical decision-support tools and research databases.

The UMLS is a comprehensive terminology integration system that acts as an “umbrella” for hundreds of biomedical vocabularies, including LOINC, SNOMED CT, and RxNorm. Rather than creating a new nomenclature, the UMLS Metathesaurus identifies equivalent concepts across different terminologies and provides a unified interface for accessing them. By mapping disparate terms to a single-concept unique identifier, the UMLS enables researchers to integrate and analyze data derived from multiple, diverse coding systems.

These systems operate jointly as the terminological backbone for health information exchange and jointly share a common data model, the Observational Medical Outcomes Partnership. In a practical clinical exchange, such as a laboratory report, LOINC is used to identify the specific test (the “question”), SNOMED CT codes the specimen source or a qualitative result (the “answer”), and UCUM provides the standardized unit for a quantitative value. This integration is supported by curated mappings between the systems—such as LOINC “parts” being mapped to SNOMED CT concepts—and their collective use is often mandated by regulatory frameworks like the United States Core Data for Interoperability to ensure true semantic interoperability [168].

LLMs can be used to extract relevant named entities from free text that translate into clinical concepts. These clinical concepts are predefined in the UMLS and can be used for neuro-symbolic processing. This process includes knowledge graphs, probabilistic mapping, and decision trees, algorithms with a more transparent and direct inner working than other, more complex deep learning approaches.

Wang et al. [169] propose a framework for early keratoconus detection that is based on both neuro-symbolic reasoning and image analysis. Relevant named entities related to clinical concepts were extracted from the free text of clinical reports. Jointly with this data, spatial morphology patterns were extracted from the report’s images. These embeddings were aligned with the symbolic representation space of the corneal knowledge graph. The model’s training was aligned with a symbolic Bayesian inference layer to produce a neuro-symbolic inference for the final diagnosis. The LLM also provides a free text explanation and treatment recommendation. The performance was near real-time, with 95 ± 15 s of runtime; the systems were tested with heterogeneous inputs, and the ground truth, knowledge graph, and final report were all evaluated by clinicians. The final report was considered clinically useful and was rated according to logical clarity, traceability of reasoning, and clinical usefulness with a 4.8 on the Likert scale. The systems obtained an AUC of 0.95 with the successful capture of subtle corneal abnormalities. The most troublesome limitation in this study was the small cohort of only 20 eyes, which is not sufficient to assess clinical safety in large-scale settings. In order to safely deploy this product, a multi-centric study with a larger dataset is required.

Miladinovíc et al. [170] propose two frameworks for retinal disease classification in OCT images. One is purely based on deep learning, and the other extracts specific features from the OCT images and uses neuro-symbolic inference—in this case, a decision tree—to obtain the final diagnosis. The neuro-symbolic approach is more transparent and yields better results.

Loaiza-Bonilla et al. [171] use LLM extraction jointly with neuro-symbolic reasoning for clinical trial matching. The LLM extraction was followed by a knowledge graph that was either directly validated or verified by a human in the loop in cases of low certainty. The knowledge graph used is aligned with SNOMED CT for interoperability.

### 8.5. Specific Approaches to LLMs

LLMs are generally categorized into attention-based, prompt-based, probing, mechanistic interpretability, causal intervention and feature attribution methods. Attention-based techniques assign importance values to individual input tokens, thereby highlighting the specific elements that most influence the model’s internal representations. In contrast, prompt-based techniques derive interpretive insights directly from the model’s generated outputs, utilizing tailored prompts to elicit explanations of the underlying reasoning processes.

An example of a neuron-activation interpretation method is the study developed by OpenAI [172], where neuron activation was linked to a certain hypothesis. In this study, the token activations in the input were compared to the tokens highlighted by a separate explanation model. This comparison can yield a score that gives some insight into the explanation model’s efficiency. The study revealed that higher layers are more abstract and provide suboptimal explanations. These methods are more easily applied to smaller models, given that for colossal LLMs, the computational costs would be too high.

Attention-based methods have even become part of explainability and LLM development in healthcare, with neuron activation being part of models applied for diagnostic procedures and directly aiding physicians [173]. Modern transformer architecture also relies on an attention-based mechanism [174]. Given the importance these systems have in modern LLM performance, explainability methods derived from them could provide relevant insight into a model’s decision. Even though this method provides some insight into the model’s inner workings, it does not completely establish a causal relationship between attention weights and causal feature importance.

An example of prompt-based methodologies is linked to the CoT mentioned in Section 3.3 Large Language Models. These explanations can be summarized by the model responsible for the textual answer and by assistant models that give explanations for the model’s decision [175]. CoT applications are related to a large improvement in medical prompting, as seen in the Medprompt application [81]. In addition to medical challenges, CoT offers improved results in several different fields. Arithmetic applications improve significantly with this method. The use of CoT not only provides a generated rationale for these black-box methods but also significantly improves their accuracy. The generated rationale may be unfaithful to the model’s internal computations [176]. These applications are especially beneficial for LLMs of a considerable size, making use of their intrinsic pre-trained knowledge.

Probing is a diagnostic interpretability technique where a supervised model, known as a probe or diagnostic classifier, is trained to predict specific linguistic properties using the feature representations from a “frozen” (without using backpropagation and with static weights) pre-trained language model to assess the type and extent of knowledge encoded within those representations. This method ranges from standard supervised classifiers to more rigorous frameworks like Perturbed Masking, Minimum Description Length (MDL), and Information-Theoretic Probing. Perturbed Masking provides a parameter-free approach that uses a two-stage masking process to estimate inter-word correlations and induce global syntactic structures. Several target words are masked, and the model generates a new representation that replaces the word in question. The distance between the original word token and the model representation is calculated through a selected metric. These distances form a heatmap that represents an impact matrix where higher values correspond to linguistic dependency or correlation between words. Graph-based algorithms are used to extract useful insights from the represented heatmap. This provides a parameter-less approach that mitigates possible bias from the trained model. Algorithms such as Eisner and Chu-Liu/Edmonds produce unlabeled dependency trees, while algorithms such as MAtRix-based Top–down parser retrieve constituent trees with a hierarchical grouping of words [177]. Information-theoretic methods further refine this by operationalizing probing as the estimation of mutual information (MI) or through MDL, which evaluates both the final quality of a probe and the “amount of effort”—measured in model size or data volume—required to achieve that quality. Complementing these, gradient-based attribution methods allow for a token-level analysis that identifies how specific representations, such as sentence-ending tokens or word-ending suffixes like “##s”, carry positional information or define distinct grammatical subspaces for features like verb tense and noun number. Practical use cases for these advanced techniques include unsupervised dependency and constituency parsing, detecting syntactic and semantic abnormalities (e.g., word-level and phrasal inversions), probing document structures through elementary discourse units, and enhancing downstream applications such as aspect-based sentiment classification (ABSC) by replacing linguist-designed dependency schemas with empirically induced trees [178].

Mechanistic interpretability is defined as the systematic study of how neural networks implement specific algorithms through their learned representations and computational structures. Moving beyond behavioral “black-box” approaches, this paradigm seeks to reverse-engineer the internal mechanisms of models to identify “circuits”—minimal subnetworks responsible for distinct algorithmic functions—and “features,” which are directions in activation space corresponding to interpretable concepts. By uncovering these internal substructures, researchers aim to move toward “white-box” alignment, enabling principled interventions such as activation steering to control model behavior or the ablation of deceptive and harmful reasoning patterns. However, the field faces fundamental challenges, such as superposition, where models pack more features than they have neurons into overlapping activations, and the difficulty of validating interpretations as models scale to increasingly complex behaviors [179].

The Medical Report Generation (MRG) survey [180] describes a transition from traditional methods such as Grad-CAM and saliency maps, which are often correlational and fail to explain complex reasoning, toward a more rigorous mechanistic interpretability framework. This paradigm shift involves reverse-engineering complex hybrid architectures, which typically integrate CNN or Vision Transformer encoders with Transformer-based LLM decoders, to uncover internal “circuits” responsible for specific diagnostic logic. By utilizing causal intervention techniques, like activation patching, and representation analysis, such as concept bottleneck probing, researchers can trace the causal flow of information from visual features directly to generated clinical tokens. Ultimately, this mechanistic approach seeks to provide verifiable, component-level explanations that align with expert medical reasoning and satisfy emerging regulatory requirements for transparency and clinical accountability.

Causal intervention is a rigorous explainability method that identifies cause-and-effect relationships within a model’s inference process by actively manipulating variables to provide explanations grounded in causal evidence. This process moves from the “low-level” circuit explanations from mechanistic interpretability towards a more holistic view of the entire process. The focus shifts from “circuits” that explain only a small part of the model to macroscopic logical reasoning that encompasses the entire model’s output, with direct, human-interpretable explanations that are usually more useful to the stakeholders involved. The conceptualization of an LLM’s decision process begins with the replacement of ground-truth labels with the LLM’s own predictions. This allows for the extraction of class-discriminative latent concepts that transform textual inputs into structured concept vectors. These structured concept vectors are modeled as a causal reasoning flow in a directed graph; this structure is validated by confirming the predictive fidelity of the identified cause in a determined model behavior. In domain-specific settings such as finance or medicine, domain knowledge can be incorporated into the retrieved graph, providing a synergistic integration that combines both paradigms into domain-specific results. Structural causal models (SCMs) leverage the causal information retrieved by the graph to perform targeted operations on specific latent concepts or intermediate hidden representations. This framework relies heavily on counterfactual reasoning: the analysis of how an output would change if an internal state or a specific input were to change. The method quantifies the causal responsibility of internal components and traces how these specific adjustments propagate to the final output. Ultimately, this framework provides explicit and verifiable explanations that reveal the reason a model reached a particular decision [181,182,183].

Bhasuran et al. [184] present the causal reasoning abilities of GPT-o1 (o1-2024-12-17) and Llama-3.2-8b-instruct using 99 clinically grounded laboratory test scenarios mapped to Pearl’s Ladder of Causation. The goal is to map clinical interventional changes and laboratory markers in a classification task and provide an explanation of the underlying rationale. The underlying rationale is evaluated by individual raters; the median of the Likert ratings for all raters comprises the final result metric. GPT-o1 demonstrated superior discriminative performance with an overall AUROC of 0.80 ± 0.12, while Llama-3.2-8b-instruct achieved an AUROC of 0.73 ± 0.15. In the reasoning evaluation, GPT-o1 achieved an AUROC of 0.76 ± 0.13, while Llama-3.2-8b-instruct scored 0.81 ± 0.01 but exhibited greater variability in its reasoning quality. Expert clinical raters favored causal explanations that traced logically consistent pathophysiological pathways. For example, associating smoking cessation with improved insulin sensitivity or reduced chronic inflammation to lower HbA1c. The models were penalized when reasoning was vague, superficial, or failed to account for clinical confounders such as age and comorbidities.

Feature attribution is directed towards the representation of a model’s explanation through the highlighting of internal components and specific input tokens that are most responsible for a generated output. In LLMs, one method of feature attribution is Conductance, which “lifts” an attribution from each input token based on the propagation of Integrated Gradients by the chain rule towards the model’s internal hidden units. This flow of attribution through specific internal hidden units allows researchers to interpret the model’s decision-making. The attributions lifted for each input have either a positive or negative connotation. The pathways associated with higher conductance are ablated, and the output of the model is checked for modifications to inspect and emphasize that these paths are the causal link behind the model’s decision. This helps developers understand if the match between specific inputs and the corresponding output has a logical binding or is simply attributed to surface-level word association [185,186].

Surrogate models are also applicable to mimic the model’s interpretations. Previously mentioned methods such as LIME and SHAP can be applicable to LLMs as interpretable models that mimic the entire LLM’s decision process. Anchor is also an available method that combines sufficient conditions, or “anchors”, within an input that locks in a model’s prediction. This process follows a sequential process: first, a specific input instance is selected to explain the associated model condition; then, the input features are systematically perturbed to observe the model changes, and a minimum set of features is selected. These features are denominated as anchors; when held constant, they ensure the prediction is maintained regardless of how the input is altered. These anchors are subjected to a high-precision threshold that provides a human-readable justification for the model’s decision [187]. This method is important in high-stakes domains like healthcare and finance because it moves beyond simple feature importance rankings to offer a rigorous guarantee of which conditions are truly necessary and sufficient for a model’s conclusion.

Ming et al. [188] present ANCHOR-RE, a pioneering neuro-symbolic and training-free framework designed to enhance the reliability of Biomedical Relation Extraction (BioRE) by bridging the gap between neural reasoning and symbolic biomedical knowledge. BioRE is an automated text-mining process that identifies and defines connections, such as protein interactions or drug–disease links, between biomedical concepts in research literature. The symbolic verification layer “anchors” and grounds the contextual reasoning of LLMs in high-precision, human-readable rules. It specifically addresses the systemic challenge of LLM over-prediction and hallucination, where models incorrectly infer meaningful relations from simple entity co-occurrences, especially in real-world datasets where the vast majority of candidate pairs are unrelated. The architecture consists of two specialized agentic modules: a Decider, which generates relation hypotheses grounded in structured evidence from external knowledge bases (such as UMLS, DrugBank [189], and CTD [190]) and retrieved in-context demonstrations, and a Verifier, which evaluates these predictions against an Error Pattern Knowledge Bank repository of reusable verification rules and recurring false-positive patterns designed to reduce model hallucinations. Experimental results across major benchmarks demonstrate significant performance gains, such as increasing the Drug–Drug Interactions Micro-F1 score from 76.9% to 87.2%, while maintaining a robust 69% precision rate on previously unseen literature published as recently as 2026. By providing a scalable, interpretable mechanism to enforce explicit semantic constraints without requiring costly model fine-tuning, ANCHOR-RE represents a practical advancement for constructing reliable biomedical knowledge graphs and supporting clinical decision-making.

TraceIn is an explainable method that quantifies the influence of individual training examples on a model’s prediction by tracing how they affect the loss of a specific test example throughout training. Inspired by the fundamental theorem of calculus, it uses gradients along the training trajectory to estimate this influence, classifying examples as proponents when they reduce the test loss (positive influence) and opponents when they increase it (negative influence). To improve scalability, TraceInCP uses saved model checkpoints and first-order gradient approximations to calculate the interaction between training and test-example gradients. TraceIn is both model-agnostic and domain-agnostic and can be applied to models trained with stochastic gradient descent or its variants. In addition to explaining individual predictions, it can identify mislabeled or anomalous training examples, find comparable examples in regression, and detect training data that may introduce unwanted correlations or biases in tasks such as text and image classification [191].

LOGRA (Low-Rank Gradient Projection) is an efficient gradient-based data valuation method designed to scale influence-function-based data attribution to billion-parameter LLMs and large datasets by avoiding the explicit computation and storage of full-dimensional gradients. It exploits the Kronecker-product structure of gradients in matrix multiplication layers, independently projecting forward and backward activations into low-dimensional spaces and directly reconstructing the corresponding projected gradients. LOGRA can be integrated into existing LLM architectures through lightweight add-on layers resembling LoRA (Low-Rank Adaptation), while the LOGIX software package, in its initial version under the Apache 2.0 license, uses PyTorch hooks to incorporate data valuation into standard training pipelines. By reducing gradient-projection complexity from *O*(*nk*) to *O*($\sqrt{nk}$), LOGRA substantially improves scalability, achieving reported gains of up to 6500× in throughput and 5× lower GPU memory usage than the Eigenvalue-corrected Kronecker-Factored Approximate Curvature influence baseline on Llama3-8B. The resulting projected gradients can also be stored and queried efficiently using vector-similarity search, facilitating repeated attribution analyses for new test examples. To preserve the most informative components during projection, LOGRA uses a PCA-based initialization informed by Kronecker-Factored Approximate Curvature, prioritizing directions associated with important curvature components and thereby retaining semantically relevant information for influence estimation [192].

## 9. Discussion

The integration of AI into healthcare presents both unprecedented opportunities and profound ethical challenges. While advanced models achieve impressive predictive accuracy, their black-box nature raises concerns regarding transparency, accountability, and trust. In clinical practice, opaque systems undermine confidence, amplify existing biases, and support spurious attribution that lack medical validity. These challenges are further exacerbated by the rapid adoption of LLMs. Although their performance on standardized benchmarks is remarkable, they remain vulnerable to adversarial manipulation that can deliberately bias outputs [89].

Addressing these risks requires a deliberate shift toward systems that prioritize interpretability, explainability, reliability, and ethical responsibility. Transparent decision-making not only fosters clinician trust but also directs predictions towards grounded clinical meaningful reasoning rather than spurious correlations. Always, of course, with the assistance of a clinician to validate the rationale provided. Moreover, explainability plays a central role in mitigating bias, safeguarding against inequities, and aligning AI systems with the core bioethical principles of autonomy, beneficence, non-maleficence, and justice.

These imperatives are echoed in international frameworks such as the EU AI Act [3] and the World Health Organization’s guidance on the ethics and governance of AI for health [91], both of which underscore explainability, inclusiveness, and protection against bias as essential requirements for responsible deployment. To illustrate this point, explainability can strengthen trust in AI-assisted diagnostic imaging, clinical decision-support tools, and predictive models for patient risk stratification. By ensuring that model reasoning remains accessible to healthcare professionals, clinicians can validate predictions against medical knowledge and context. Moving forward, healthcare AI research and implementation must strike a balance between performance and transparency, ensuring models avoid misleading outputs, minimize reliance on counterfactual explanations, and contribute to fair, unbiased, and ethically sound decision-making.

The EU AI Act enforces the core principles of biomedical ethics. Multiple Articles enforce the requirement for transparency, control, and bias mitigation, which resonates greatly with the principle of justice in biomedical ethics. Additionally, the explainability and transparency from these frameworks enhance the patient’s autonomy and decision-making with clearer boundaries.

Article 9 ensures the implementation of a continuous risk assessment that protects vulnerable groups, while Article 10 ensures the high-quality of datasets through comprehensive data lifecycle management practices, including error-free, representative, contextualized data, with mandatory, secure bias mitigation measures. Both these principles ensure that justice is present within the development and deployment of AI-related methods in healthcare. All groups within society should have equity in medical treatment. Misuse and irresponsible utilization of AI can further enlarge the already established differences present within the status quo. Assurance of these Articles through bias mitigation is not only a matter of justice, but of implementation of non-maleficence practices to avoid the unfair mistreatment of minorities and promote the betterment of autonomy within a patient’s decision-making.

Article 13 of the AI Act requires that high-risk AI systems provide sufficient transparency to enable deployers to appropriately interpret and use system outputs. This requirement is primarily fulfilled through information concerning the system’s capabilities, performance characteristics, limitations, intended purpose, and instructions for use, rather than through mandatory explainability mechanisms or complete reproducibility of individual model decisions. Article 14 relates to human oversight in model deployment. This human-in-the-loop approach is crucial to shaping an effective model. Article 15 ensures that models are robust and reliable, enforcing the need for accurate and efficient models that effectively contribute to healthcare. The principles in these Articles are symbiotic; higher efficiency from the model requires transparency and human oversight. Transparency gives insight into a model’s decision, validating the model’s results and making them humanly interpretable, which results in more reliable and robust outcomes. Visual explanations are a crucial point in diagnostic interpretation and aid in providing transparency and reliability to the model’s decision. The technical procedures discussed above, saliency maps and heatmap methods, are part of ensuring that the legislation is abided by and the biomedical principles are upheld.

The variety of available explanation methods can provide effective diagnostic support for a wide range of possible models. The use of gradient- or activation-based visual explanations can be adjusted accordingly. Activation-based methods such as CAM, upgraded methods from CAM, Grad-CAM, CGBP, and LRP provide a range of options for model explainability visualization. These activation-based visualizations highlight specific regions of the image and are more blurred and sparser.

In contrast, gradient-based methods such as saliency maps, RectGrad, and Guided Backprop provide explanations reliant on edges and are less sparse. A combination of both these approaches can enhance the model prediction’s explanation and improve transparency. The combination of visualizations exhibited from both these explanations results in a detailed outline of the underlying model’s inner workings behind the decision process.

Model-agnostic methodologies, such as SHAP and Shapley, can be relied on to present possible important predictors in tabular data. Meanwhile, methodologies such as LIME and RISE present an occlusion-based method in image visualization, which is relevant regardless of the underlying model. This presents an opportunity for application across a wide range of models, regardless of their underlying technology.

Neuro-symbolic-based methods provide a transparent and clear approach to healthcare. The combination of deep learning accuracy with the clarity and transparency of neuro-symbolic reasoning results in clear decision-making grounded in concrete knowledge-based systems and evidence. This process makes the decision-making rule-based and evidently transparent, with simplification from complex deep learning approaches to transparent white-box models. The application of an LLM also has some disadvantages, such as LLM extraction errors, incomplete ontologies, incorrect entity linking, and biased symbolic rules, that may propagate through the system.

These processes are especially efficient with LLMs. The use of LLMs for named-entity extraction followed by neuro-symbolic reasoning provides a transparent, evidence-based system with the robustness and accuracy associated with an LLM’s performance.

The incorporation of varied explainability methods is paramount; however, an excessive number of variables related to a clinician’s decision can lead to cognitive overload. On the other hand, relying simply on one method limits the explanation’s fidelity, giving room for misleading explanations to easily deceive the physician. A robust explanation relies on more than one input. The simultaneous incorporation of these methods must be complementary and incisive to balance this trade-off correctly. For example, an implementation that focuses on analyzing how relevant each input is for the observed output, in conjunction with a method that verifies the causal relation between the most relevant inputs and the model’s prediction.

The principles of biomedical ethics, such as autonomy, beneficence, non-maleficence, and justice, require transparency. The embodiment of these principles is present in legislation such as the EU AI Act.

From deep learning black boxes to simpler white-box methods that provide explanations from low-complexity, transparent processes, there are abundant case studies and methods available that provide the transparency required for the EU AI Act.

Compliance with the EU AI Act is a step forward towards obtaining the CE Mark. In Europe, the CE Mark is the crucial milestone to achieve regulation compliance. As such, the implementation of reliable and adequate explanation methods is paramount for market entry.

The strict regulations enforced by the EU constitute a crucial step in safeguarding our most personal and sensitive healthcare data. Legislation that mitigates cybersecurity threats, such as NIS2, CRA, and the cybersecurity solidarity act are crucial to maintaining our personal data private and safe.

Given the growing impact of AI in healthcare settings, there is a growing concern about developing XAI practices that are relevant and well-adjusted. The development in these approaches ranges from image-based models and multimodal models to LLMs. The combination of technically relevant, ethically sound, and lawful AI deployment is of utmost relevance to this topic. A proper deployment of AI models results in benefits for multiple stakeholders: patients, doctors, and hospital management.

Patients benefit from improved healthcare with greater performance and efficiency in both treatment and diagnosis. This improved healthcare rests on more grounded, explainable decisions.

Doctors also benefit from grounded, explainable decisions for medical diagnosis in their practice. These approaches mitigate possible clinical errors. Although an exaggerated and redundant implementation of explainability methods can lead to cognitive overload, overall, a well-adjusted application of explainability methods that mitigate biases and errors has positive results.

This review has several limitations that should be considered when interpreting its findings. First, the source-selection process may not be fully reproducible, as the search and inclusion procedures may have involved subjective decisions. The restriction to English-language sources may also have introduced language bias and resulted in the omission of relevant evidence published in other languages. Furthermore, no formal quality appraisal of the included sources was performed, limiting the ability to assess differences in methodological rigor and evidential reliability. The inclusion of heterogeneous source types, ranging from peer-reviewed studies to technical reports, guidelines, and legal or regulatory documents, further complicates direct comparison of the evidence. In addition, the rapidly evolving nature of AI research means that reliance on dynamic benchmarks and continuously changing technical standards may affect the stability and long-term validity of some conclusions. Publication bias cannot be excluded, particularly because positive or novel findings may be more likely to be reported, while potential self-citation bias may have influenced the representation of particular research groups or perspectives. Finally, the rapid obsolescence of technical developments, benchmarks, regulatory frameworks, and legal material presents an important temporal limitation, meaning that some conclusions may require reassessment as the underlying technologies, standards, and regulatory landscape continue to evolve.

Explanation fidelity is an important consideration when evaluating XAI methods, as an explanation that appears clinically plausible does not necessarily reflect the model’s actual decision-making process. In healthcare, visually intuitive heatmaps or feature attributions may highlight clinically relevant regions while failing to accurately represent the features that causally or functionally drive the model’s prediction. Therefore, clinical plausibility should be distinguished from faithfulness or fidelity, which concerns the degree to which an explanation corresponds to the model’s underlying decision function. Fidelity can be assessed using model-based approaches such as perturbation or deletion tests, in which features identified as important by the explanation are systematically removed or altered, and the resulting change in the model’s output is measured. Additional approaches include sensitivity analyses and comparisons between explanations and model responses to controlled input changes. Incorporating such evaluations would strengthen the manuscript by demonstrating that the reported explanations are not only clinically interpretable but also reliably represent the information used by the model to generate its predictions.

Explanation stability is another important but underdeveloped aspect of XAI evaluation. An explanation should ideally remain reasonably consistent when factors that do not fundamentally alter the model’s decision are changed. However, explanation outputs may vary substantially because of different random seeds, perturbation procedures, baseline or reference samples, preprocessing pipelines, or small changes to the input data. This issue is particularly relevant for attribution methods applied to correlated predictors, where importance may be distributed differently among highly correlated features without substantially changing the model prediction. Consequently, an explanation may appear precise while being sensitive to arbitrary methodological choices rather than reflecting a robust property of the model. Explanation stability can therefore be assessed by repeatedly generating explanations under controlled variations and quantifying their similarity using measures such as rank correlation, intersection-over-union, attribution correlation, or structural similarity, depending on the explanation modality. Reporting such sensitivity analyses would strengthen the evaluation by demonstrating whether the identified features or regions consistently contribute to predictions rather than representing artifacts of a particular random seed, preprocessing choice, reference sample, perturbation strategy, or minor variation in the input data.

High model-performance metrics such as AUC, accuracy, sensitivity, and specificity should not be conflated with XAI-specific metrics such as faithfulness, stability, interpretability, and clinical usefulness, as strong predictive performance does not necessarily indicate that an explanation accurately reflects the model’s decision process or provides reliable clinical insight. This further emphasizes the need for explainability solutions that grant fidelity to a model’s prediction and incorporate these metrics into the legislative process.

Explainability should be evaluated through measures of faithfulness, stability, and clinical usefulness. Faithfulness assesses whether an explanation reflects the model’s actual decision function and can be quantified using perturbation-based measures. Insertion and deletion curves provide related assessments, with higher insertion AUC and lower deletion AUC generally indicating greater faithfulness. Explanation stability evaluates whether similar explanations are produced under small changes in the input or explanation procedure and may be quantified using measures such as Spearman correlation or intersection-over-union across explanations generated with different random seeds, perturbations, reference samples, preprocessing procedures, or correlated predictors. Clinical usefulness evaluates whether explanations provide measurable benefit to clinical users and can be quantified as ΔPerformance = Performance_clinician+XAI_ − Performance_clinician_, where the performance is represented through a clinical decision-performance measure such as diagnostic accuracy, sensitivity, or specificity. Together, these measures distinguish whether an explanation reflects the model’s reasoning, remains robust, and provides practical clinical value, rather than if high model performance or visual plausibility alone demonstrates effective explainability.

The implementation of these metrics requires solid causal bindings between the model’s predictions, the generated explication rationale, clinical knowledge, and a clear causal relation between the explication rationale and the provided prediction. The implementation of these measures must go beyond a simple heatmap implementation. This can be achieved through ablation methods, counterfactual explanations, or other approaches that clarify this causal binding further than a mere correlation.

Patient-facing explanations require particular consideration because explanations designed for clinicians may not be appropriate for patients with different levels of health literacy, accessibility needs, or preferences. Complex model outputs should be communicated in understandable language and formats while minimizing cognitive burden, particularly when patients are confronted with uncertain, probabilistic, or technically complex information. Explanations may also have emotional effects, potentially increasing anxiety when highlighting disease-related risks or, conversely, creating false reassurance when an AI prediction appears highly confident despite underlying uncertainty. Patient-facing XAI should therefore adopt accessible and user-centered explanation strategies that account for differences in health literacy, cognitive needs, emotional responses, and individual preferences.

The incorporation of specific approaches within the regulatory framework is key to achieving transparent and trustworthy AI implementation in healthcare. Beyond general requirements, explainability should be explicitly linked to regulatory obligations. As shown in Table 3, visualization methods, audit trails, explanation reports, and human oversight mechanisms can provide concrete evidence of compliance with Articles 11–15 of the EU AI Act. Such a framework would facilitate both regulatory assessment and the safe deployment of AI systems in clinical practice.

Overall, the investment in AI-related solutions and deep learning deployments in healthcare benefits multiple stakeholders and results in both cost reduction and performance improvement for diagnostics, prognostics, and treatment planning [193]. A case study from Tongji Hospital has shown an improvement of 6% in Alzheimer’s diagnostic accuracy and an average reduction in assessment time of 37 min [4]. This represents the potential that AI implementation has in healthcare.

## 10. Conclusions

Ultimately, advancing trustworthy, fair, accountable, and transparent AI in healthcare depends on the continued refinement and evaluation of explainability methods. A diverse range of techniques already exists, but sustained progress will be essential to strengthen reliability, enhance clinician confidence, and ensure that AI evolves into a truly trustworthy partner in improving patient care. Thus, integrating explainability is not merely a technical requirement, but a clinical and ethical imperative for the future of AI in healthcare—even with the possibility of unstable, misleading, or cognitively burdensome explanations. These concerns must be mitigated with the presence of a clinician who provides assistance and an overview of the entire process.

All stakeholders—patients, doctors, and hospital management—can benefit from improved AI deployment and more transparent automated decision-making processes. Explainability-based methods are an essential part of this process to make decisions more transparent and trustworthy.

## Figures and Tables

**Figure 1:**
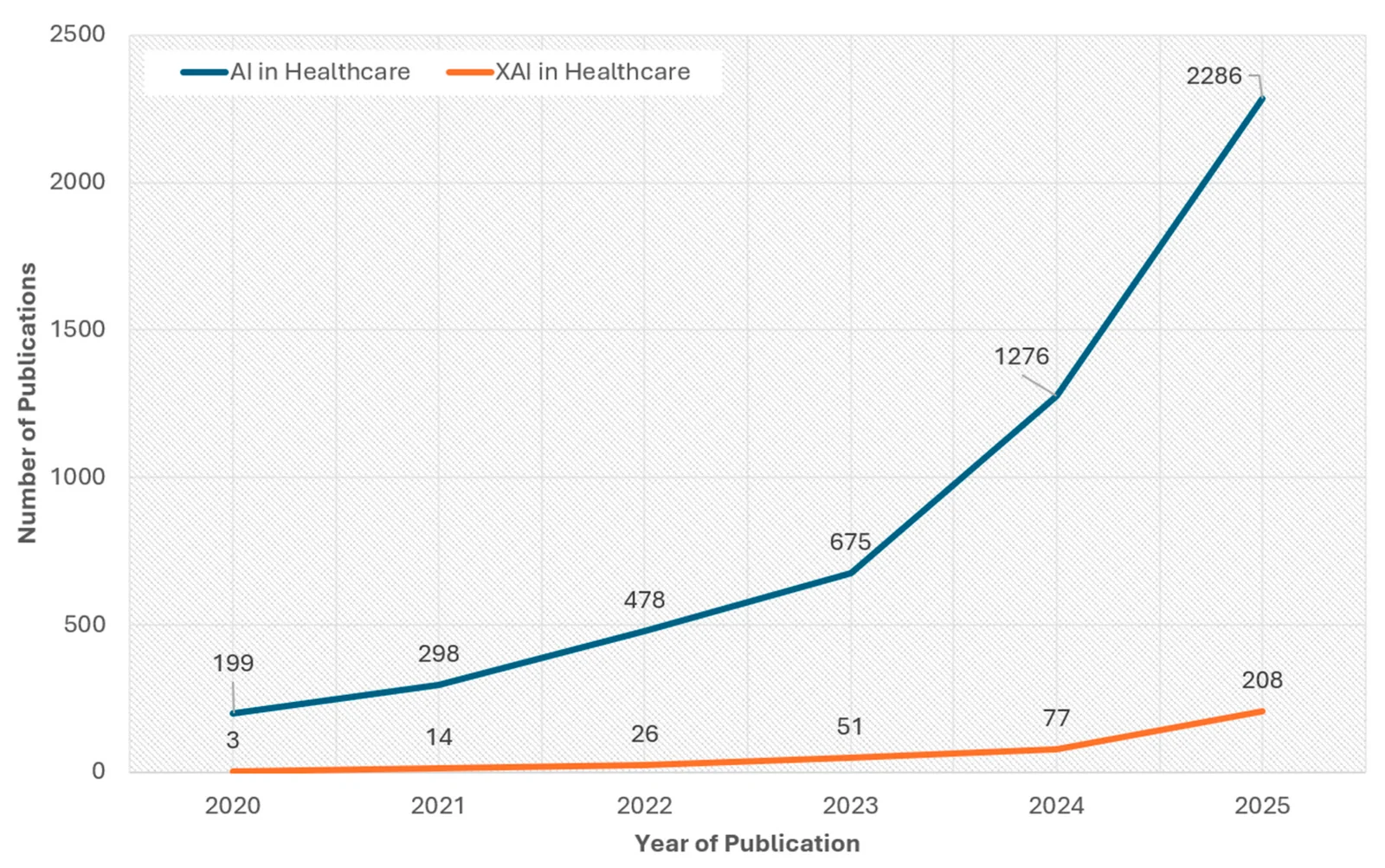
Trend of yearly publications on AI in healthcare (2019–2025).

**Figure 2:**
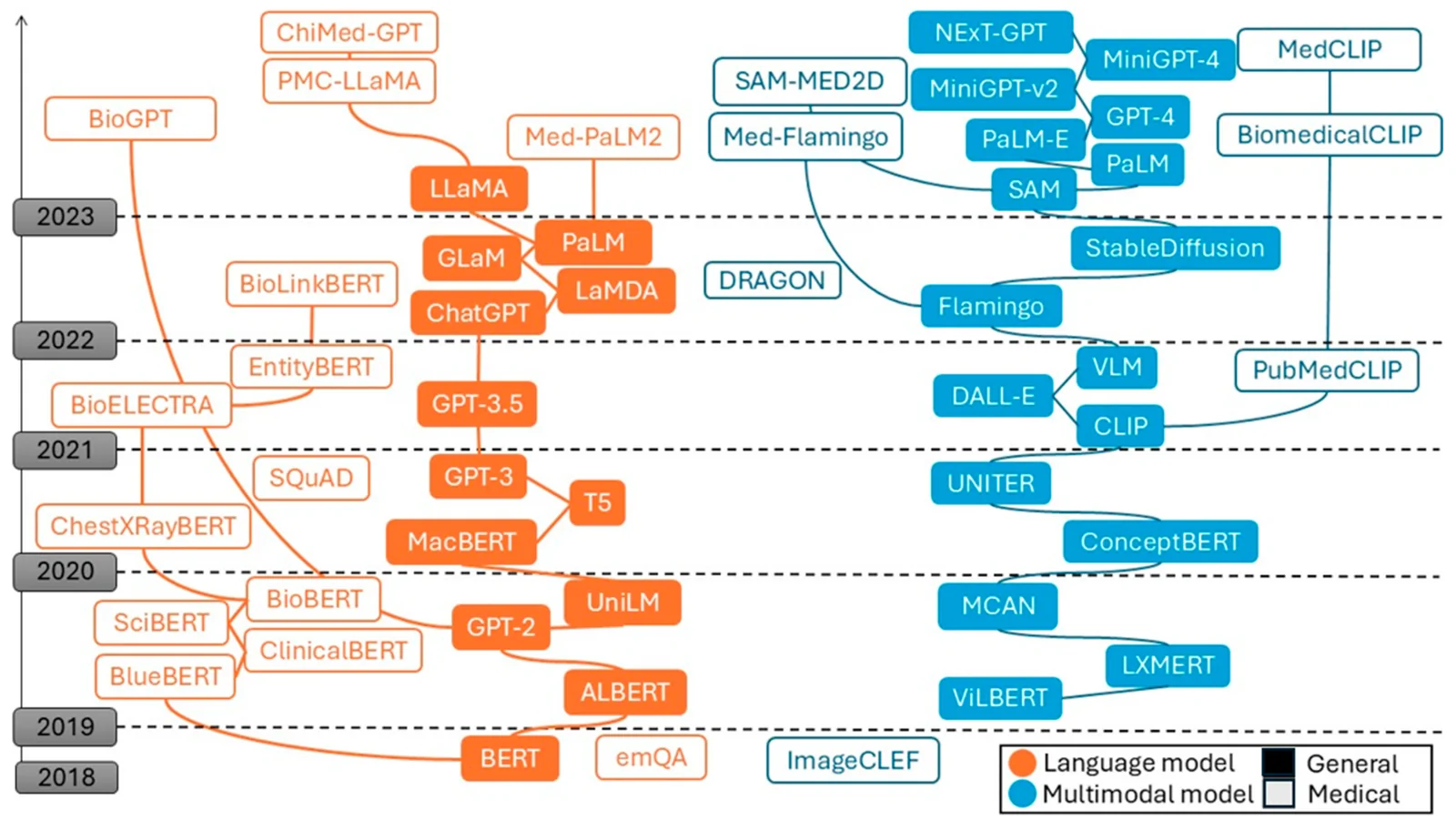
Evolution of simple and multimodal LLMs until 2024. Adapted from [47].

**Figure 3:**
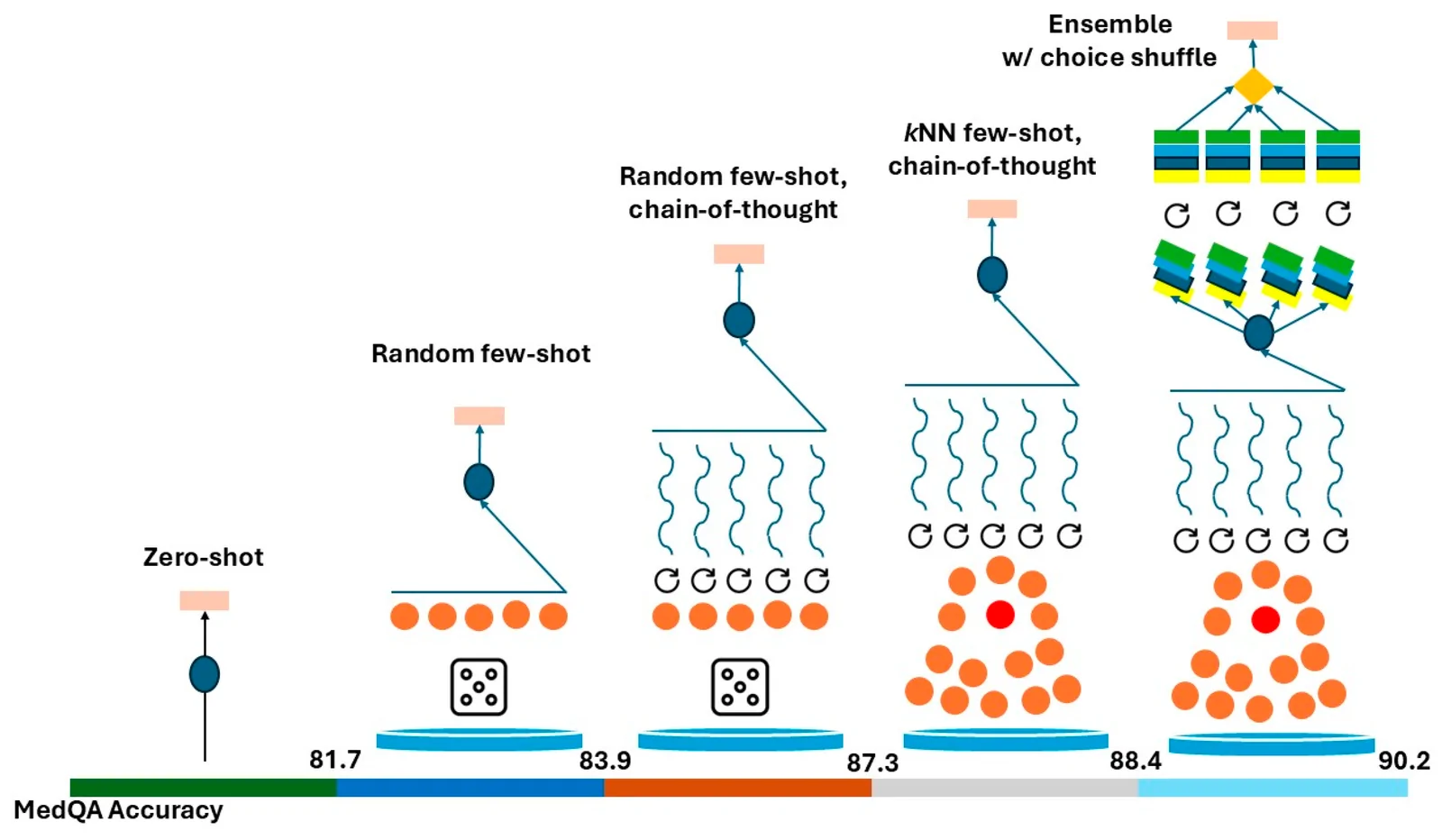
Step-by-step Medprompt explanation. Adapted from [81].

**Figure 4:**
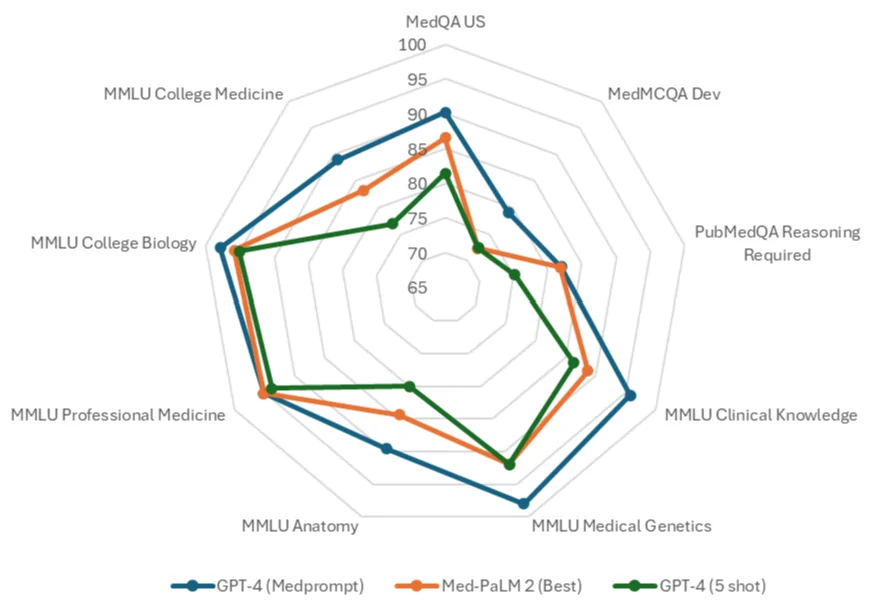
ChatGPT-4 with Medprompt, Med-PaLM 2, ChatGPT-4 (5 Shot) benchmark performances. Adapted from [81] (November 2024).

**Figure 5:**
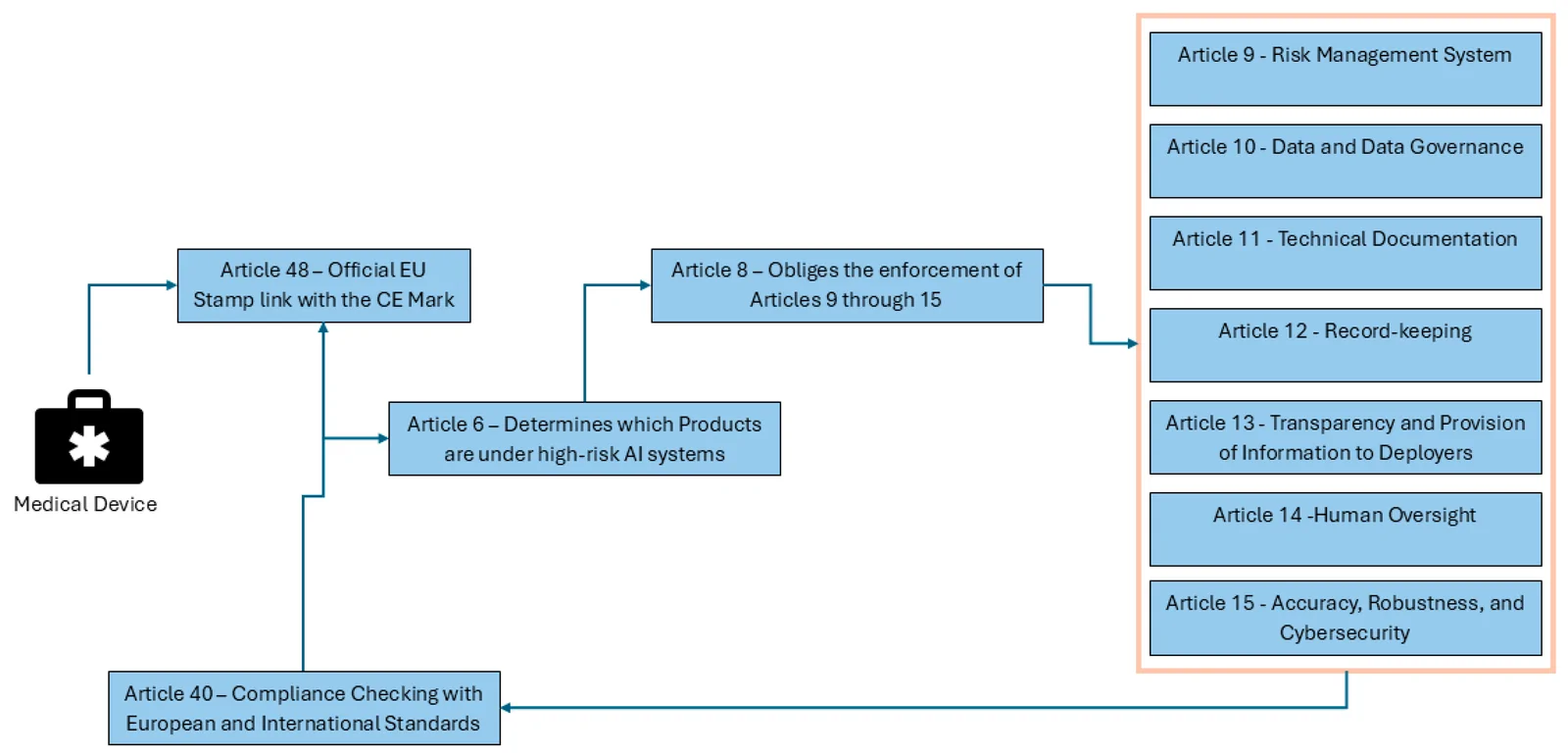
EU AI Act high-risk device implementation diagram.

**Figure 6:**
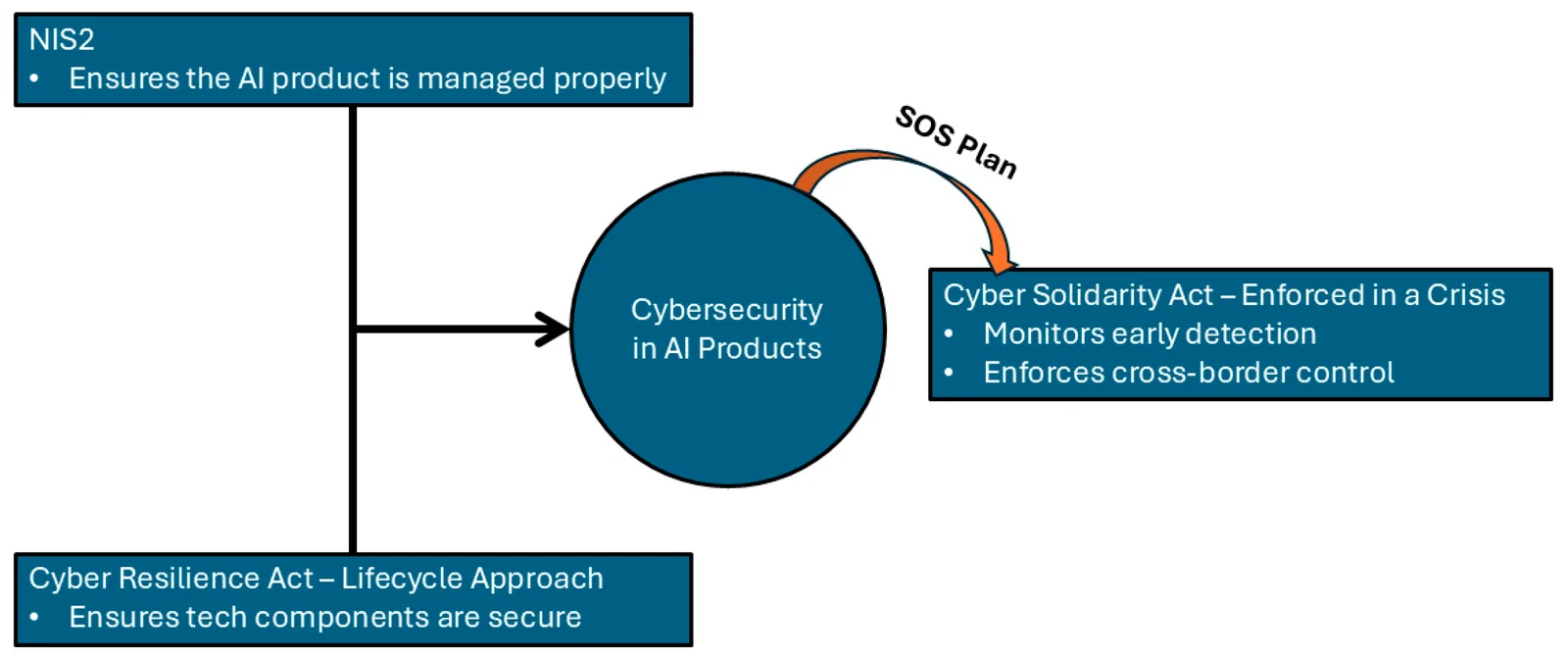
Cybersecurity requirements in the EU.

**Figure 7:**
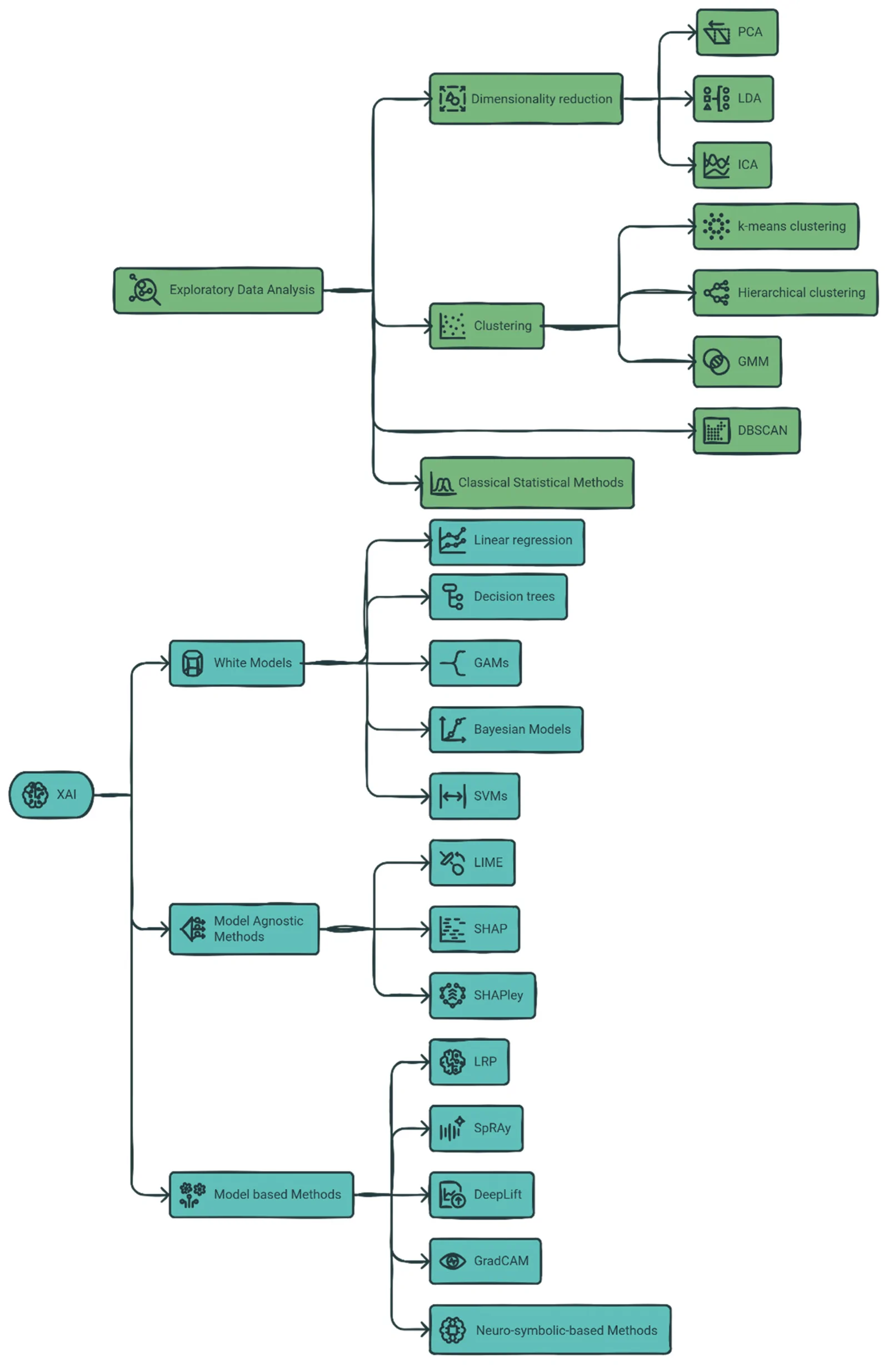
Taxonomy of exploratory data analysis and explainable artificial intelligence (XAI) approaches. XAI methods are grouped into four main categories: Exploratory Data Analysis, which includes dimensionality reduction (Principal Component Analysis, PCA; Linear Discriminant Analysis, LDA; and Independent Component Analysis, ICA), clustering (Gaussian Mixture Models, GMM; Density-Based Spatial Clustering of Applications with Noise, DBSCAN), and classical statistical techniques; White Models, which are inherently interpretable models such as linear regression, decision trees, Generalized Additive Models (GAMs), Bayesian models, and Support Vector Machines (SVMs); Model-Agnostic Methods, which provide explanations independently of the underlying model architecture, including Local Interpretable Model-Agnostic Explanations (LIME), SHapley Additive exPlanations (SHAP), and SHAPley-based approaches; and Model-Based Methods, which generate explanations from the internal structure of machine learning models, including Layer-wise Relevance Propagation (LRP), Spectral Relevance Analysis (SpRAy), Deep Learning Important FeaTures (DeepLIFT), Gradient-weighted Class Activation Mapping (Grad-CAM), and neuro-symbolic approaches.

**Figure 8:**
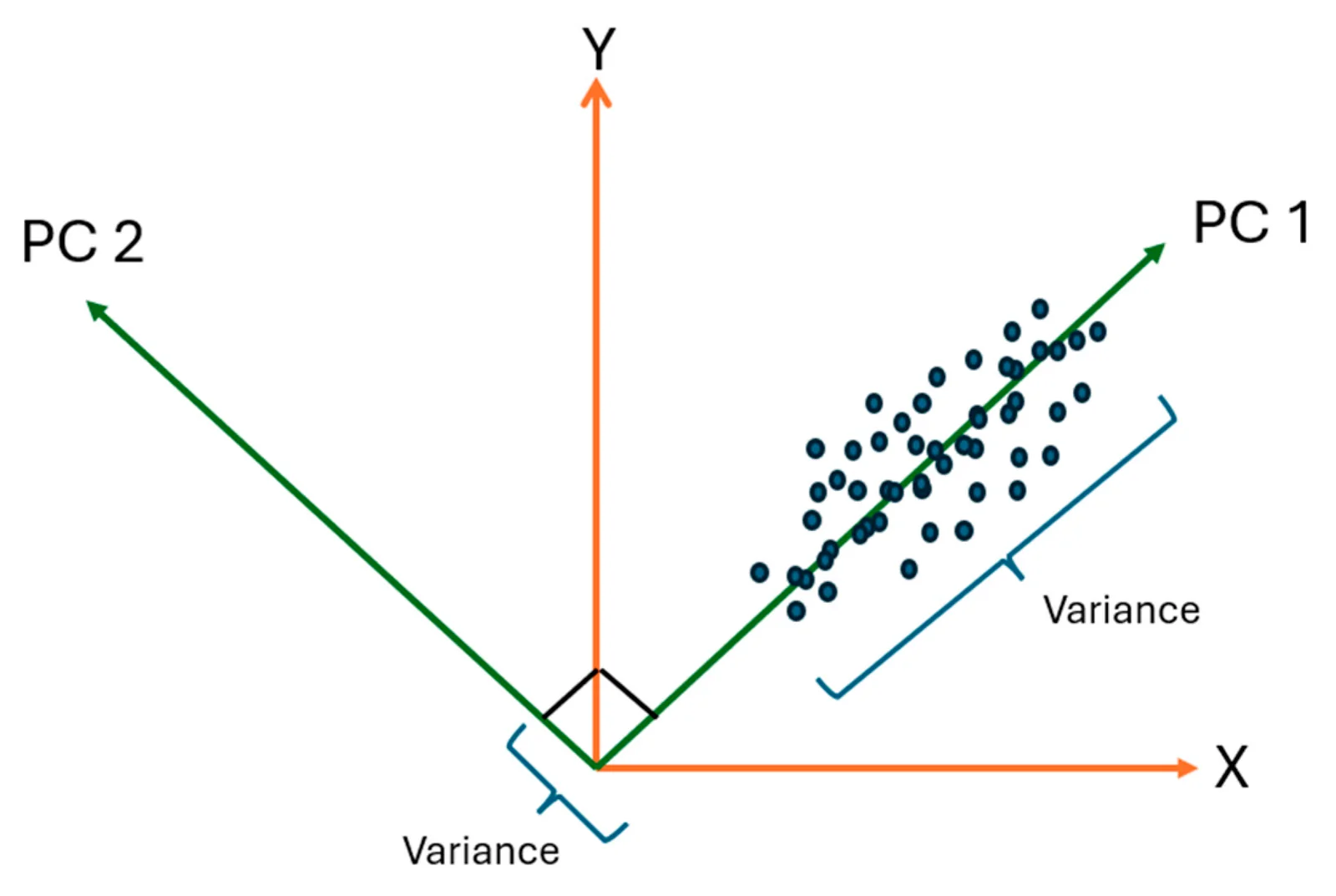
Principal component analysis eigenvector representation, the PC stands for principal component.

**Figure 9:**
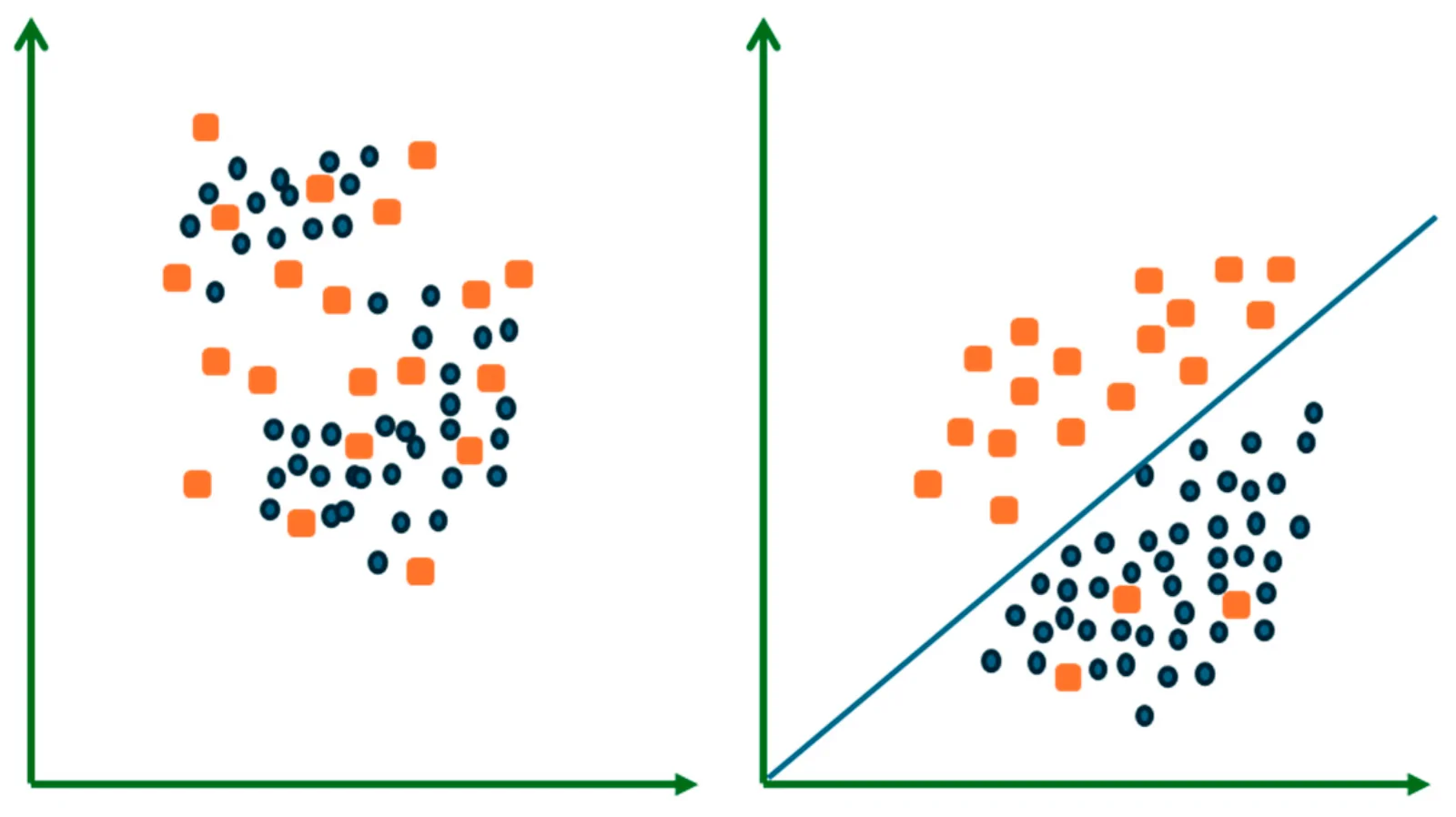
Linear discriminant analysis application.

**Figure 10:**
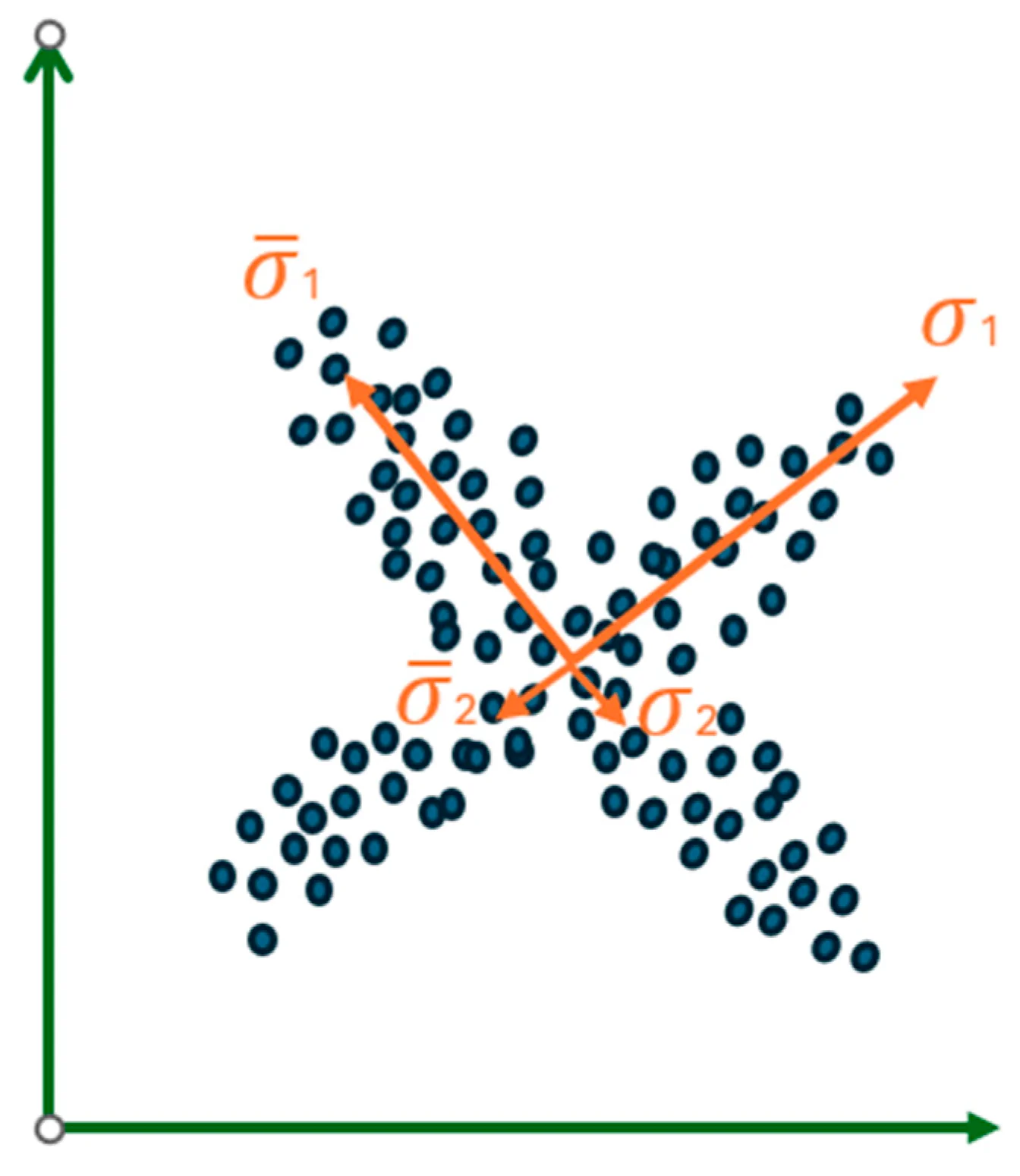
Independent component analysis vector visualization, σ1 and σ2 represent the two independent component vectors, while $\overset{-}{\sigma }1$ and $\overset{-}{\sigma }2$ represent their orthogonal vectors.

**Figure 11:**
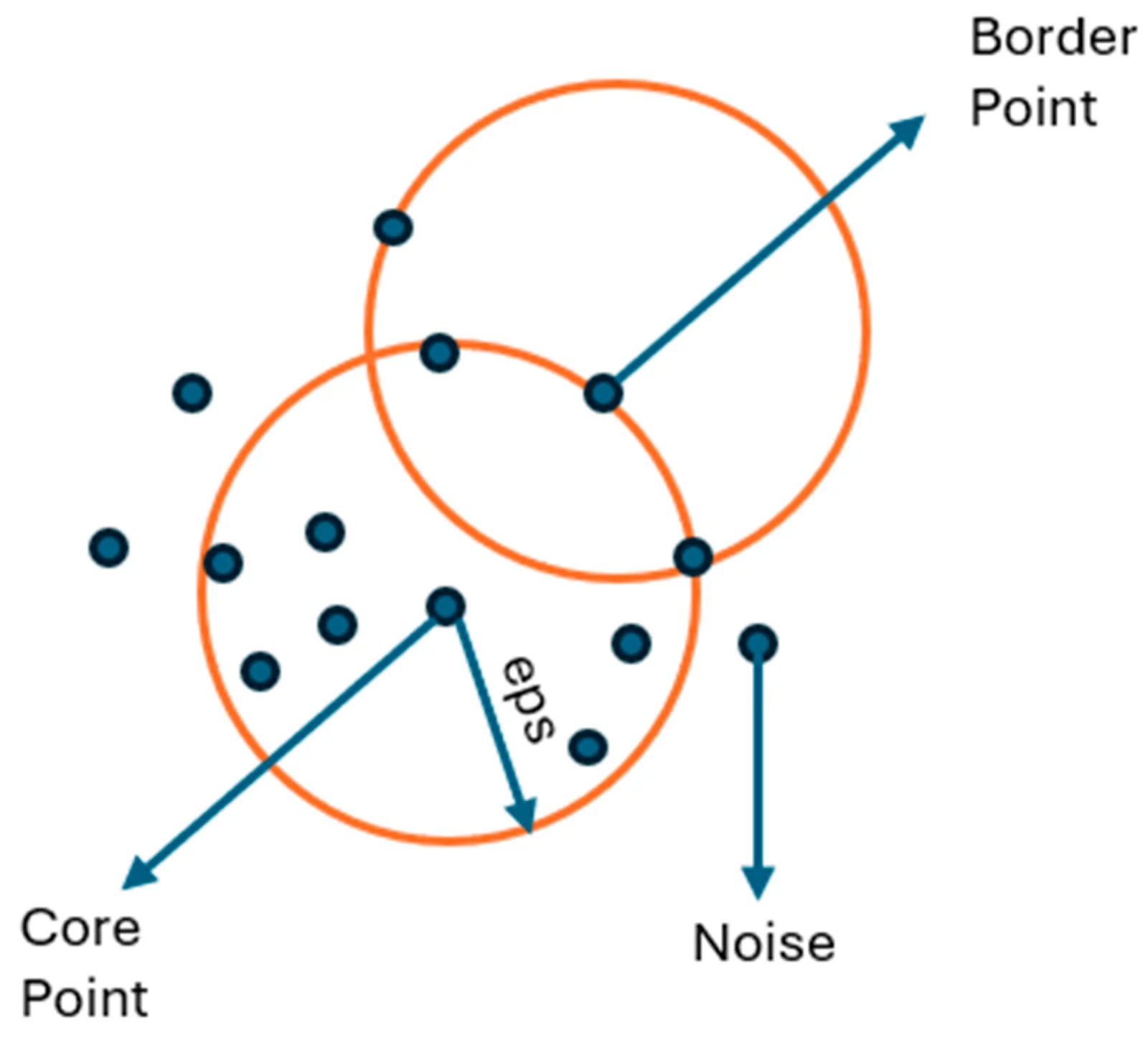
Visualization of DBSCAN clustering algorithm. Adapted from [114].

**Figure 12:**
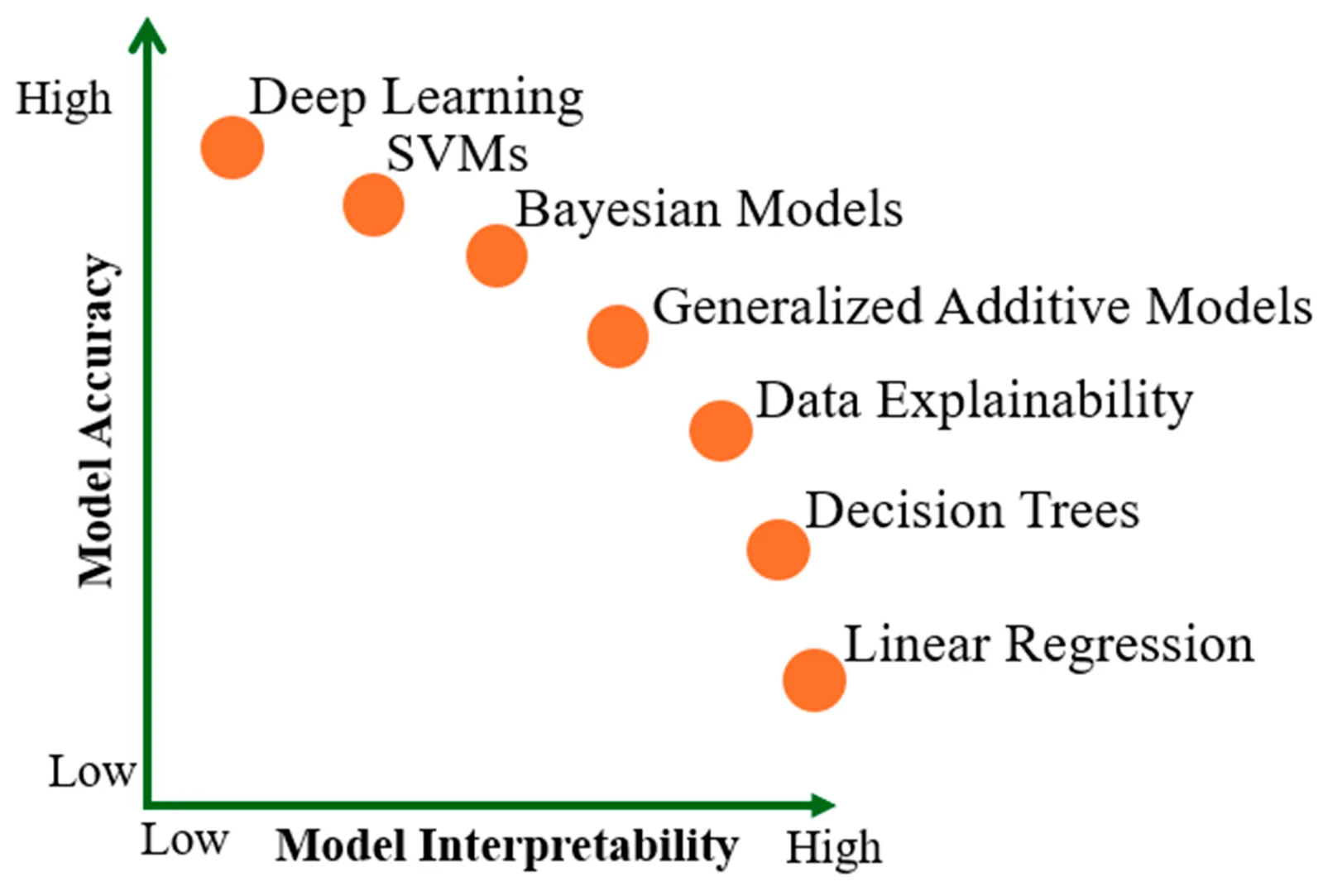
Trade-off between model accuracy and interpretability.

**Figure 13:**
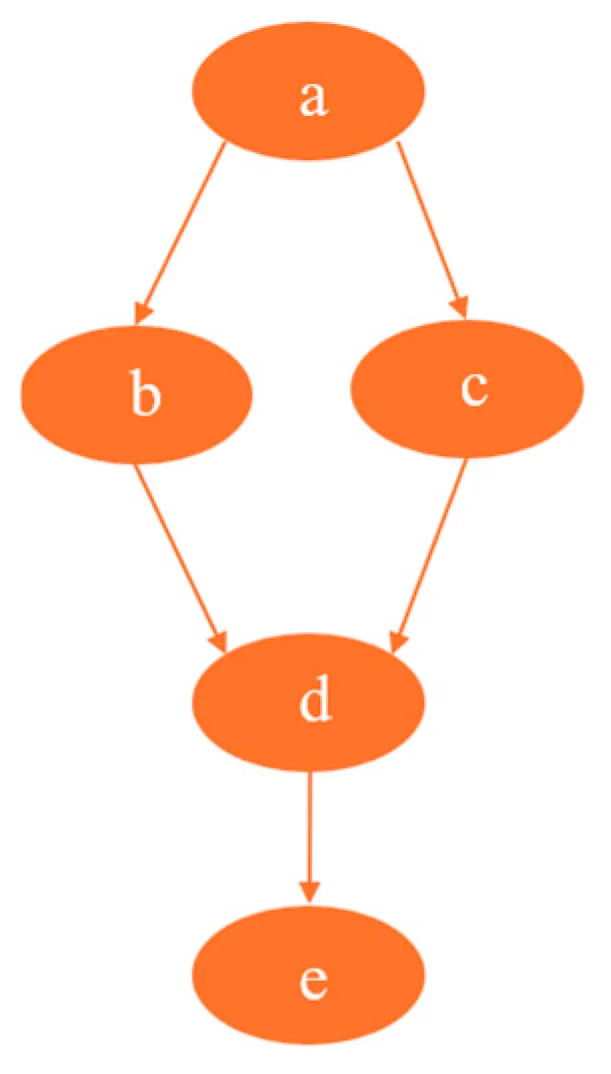
Directed acyclic graph representation. Adapted from [124].

**Figure 14:**
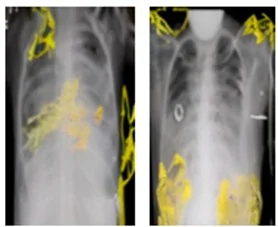
LIME application in viral pneumonia detection: yellow contours correspond to the *superpixels*, which are LIME features most relevant for the model’s decision. Adapted from [136].

**Figure 15:**
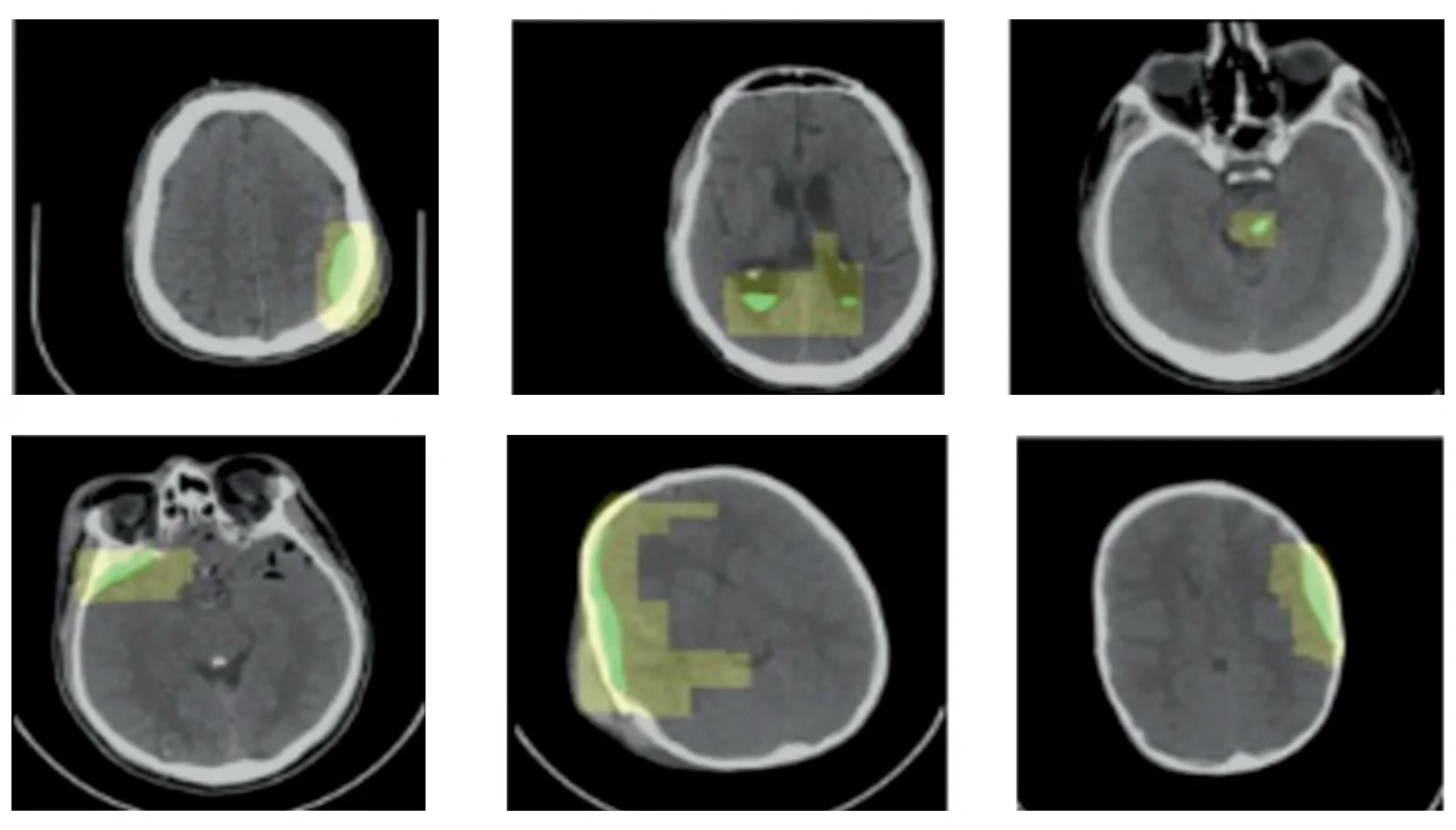
3DCT images from brain hemorrhage detection: green marks indicate the bleeding ground truth, and yellow marks correspond to RISE. Adapted from [141].

**Figure 16:**
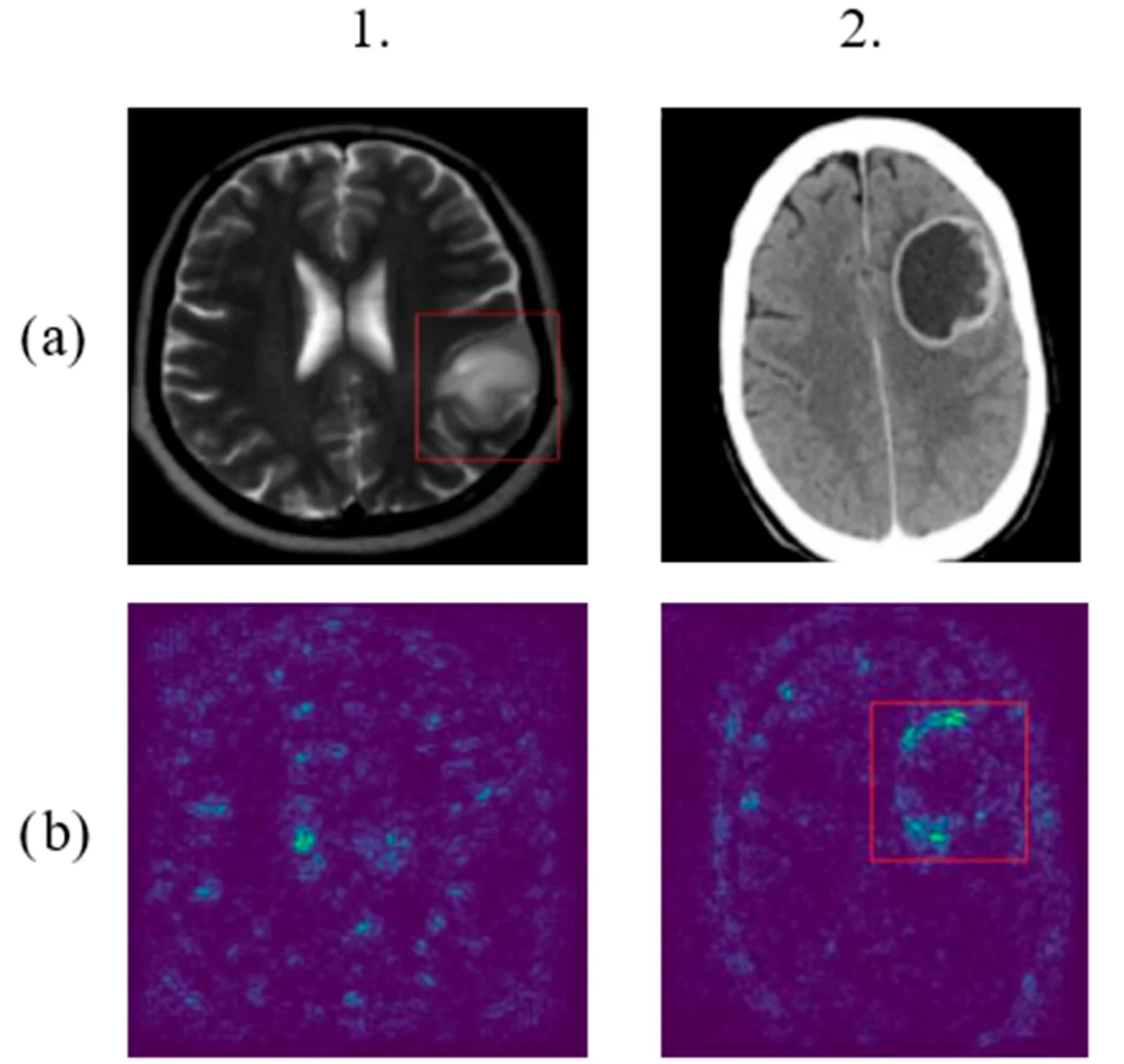
MRI image and respective saliency map: (**a**) MRI image, (**b**) saliency map, **1**. correctly classified, **2.** incorrectly classified, the red square signifies tumor location. Adapted from [146].

**Figure 17:**
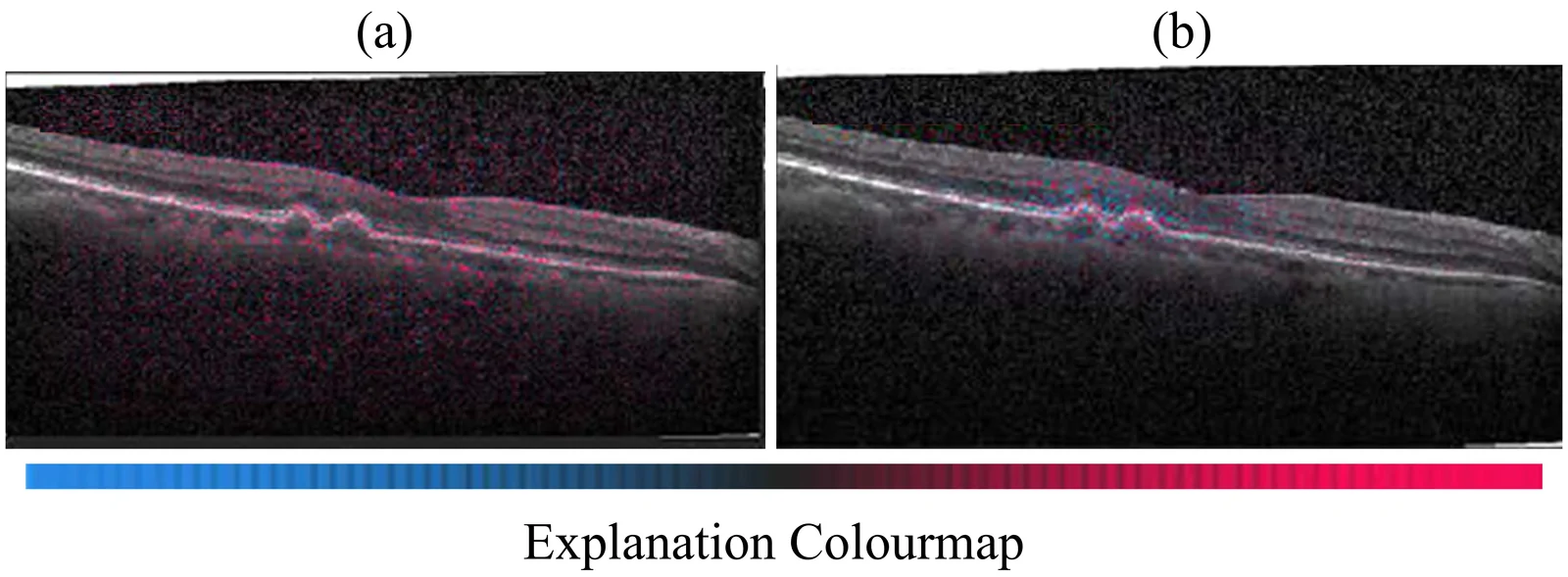
Two different visual representations of explanation methods, with a color map from blue to red related to OCT Drusen classification: (**a**) DeconvNet and (**b**) SmoothGrad explanation examples. Adapted from [150].

**Figure 18:**
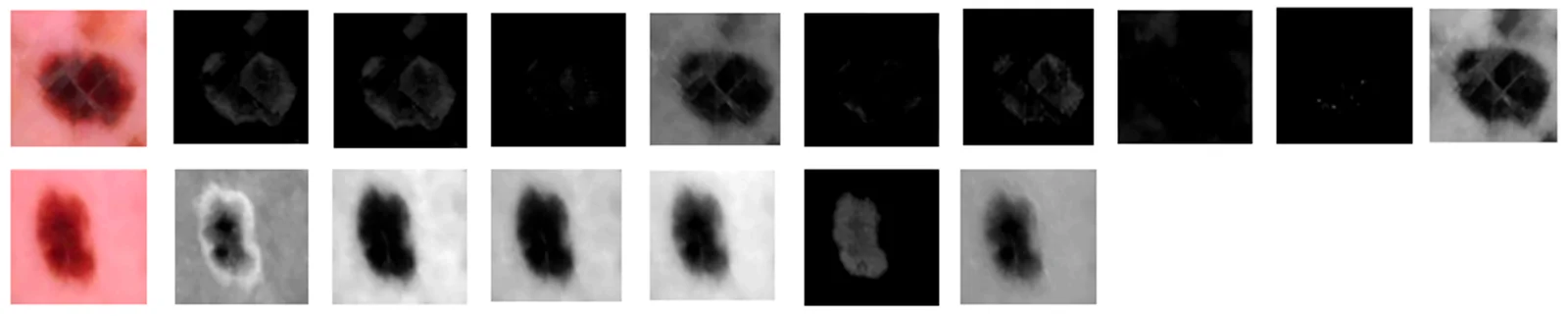
Sequential feature maps from two different input images. Adapted from [153].

**Figure 19:**
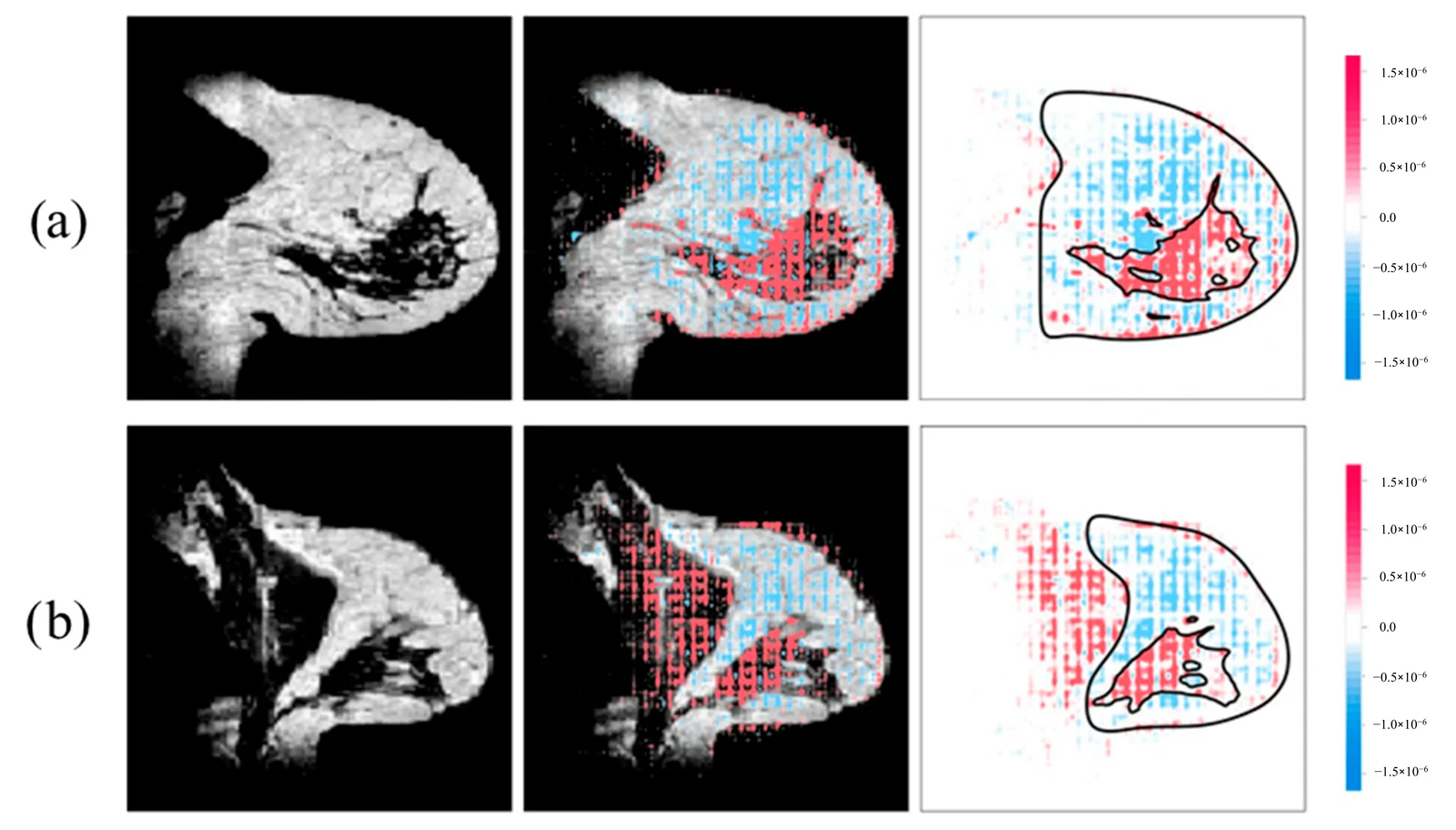
Deep SHAP map superimposed on MRI images: (**a**) correctly classified images, (**b**) incorrectly classified images. The (**left**) image is a sagittal slice of the precontrast MRI, the (**middle**) image is the same sagittal slice with the SHAP map superimposed, and the (**right**) image is the corresponding SHAP map with boundaries of the breast and fibroglandular tissue shown in black contours. Adapted from [17].

**Figure 20:**
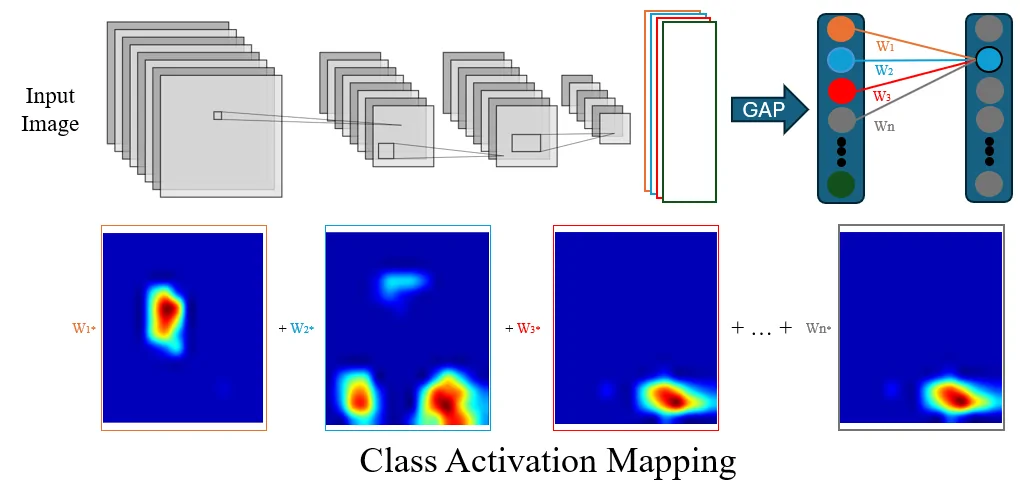
Scheme of Class Activation Mapping application, each colour represents one set of weights, the * symbolizes multiplication between each weight and feature map, the ellipses indicate the missing weights from the image representation comprised between weight number 3 and the last weight. Adapted from [156].

**Figure 21:**
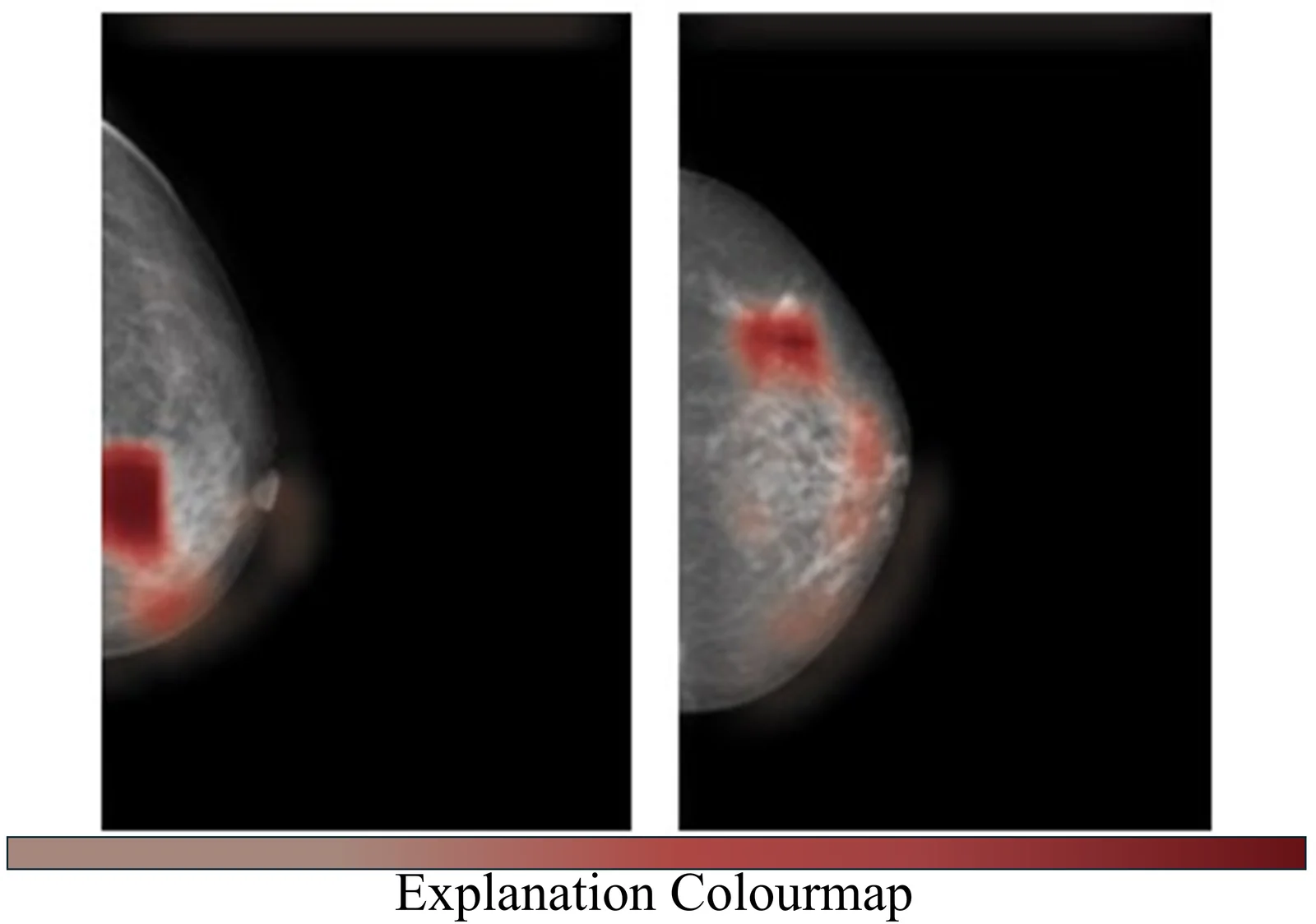
Class Activation Mapping in breast lesion detection. Adapted from [157].

**Figure 22:**
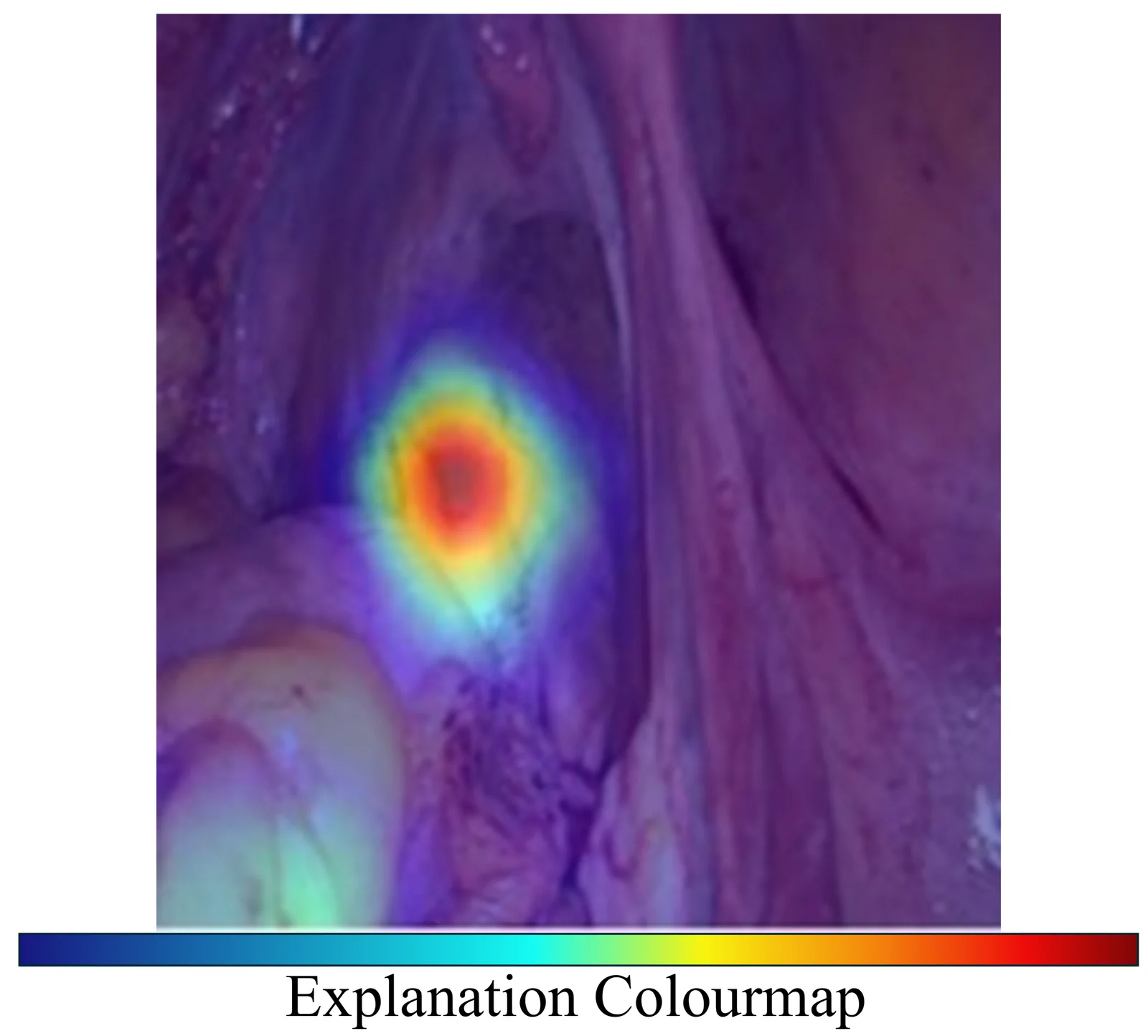
Grad-CAM generated heatmap for anastomosis prediction. Adapted from [159].

**Figure 23:**
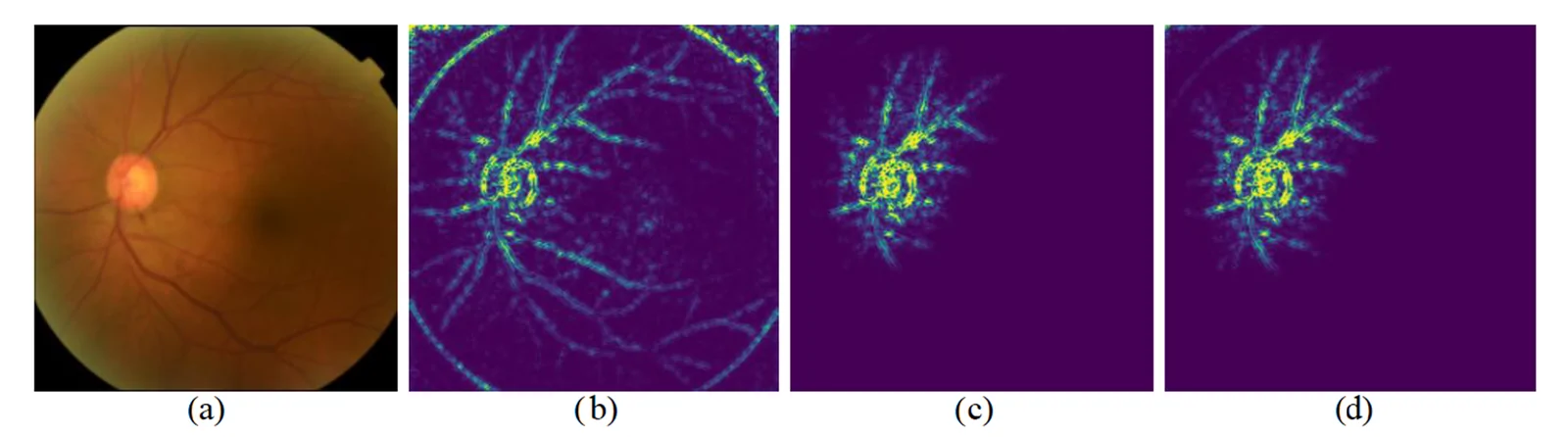
Retinography images from a glaucoma patient: comparison between the Guided Backprop (**b**), RectGrad (**c**), and CGBP (**d**) explanation methods; (**a**) represents the original image. Adapted from [156].

**Figure 24:**
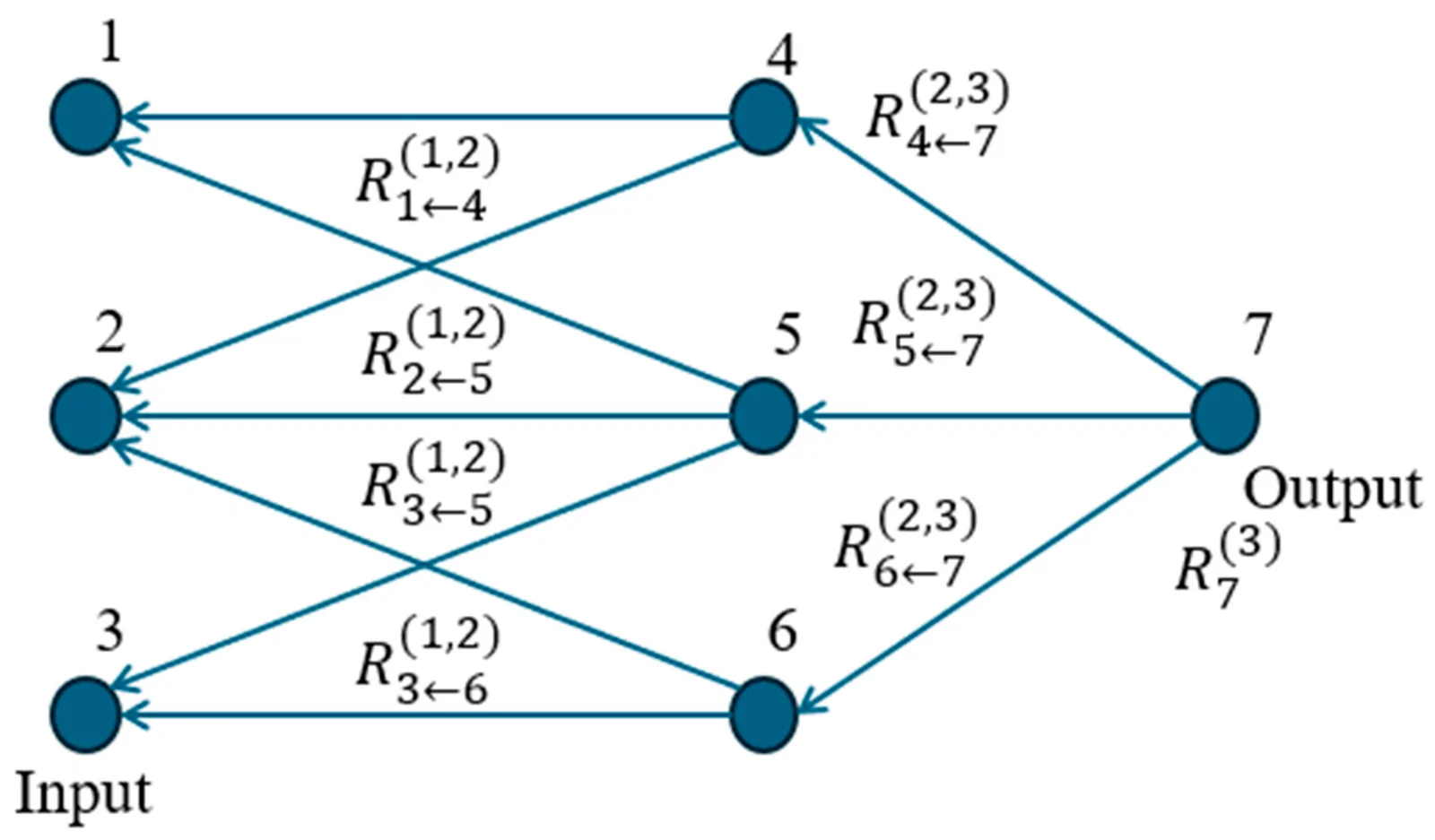
Relevance mapping backward from the output target neuron. Adapted from [161,162].

**Table 1:** LLMs’ accuracy in the MedQA dataset [82] from the 14th of August 2026.

Model/Model Version	Accuracy in the MedQA Dataset (%)	95% CI
o1/openai_o1-2024-12-17	96.52%	±0.33
GPT-5.1/openai_gpt-5.1-2025-11-1	96.38%	±0.17
Gemini 3.1 Pro Preview/google_gemini-3.1-pro-preview	96.37%	±0.17
GPT-5/openai_gpt-5-2025-08-07	96.32%	±0.17
GPT-5.4 (xhigh)/openai_gpt-5.4-2026-03-05	96.09%	±0.18
o3/openai_o3-2025-04-16	96.06%	±0.18
GPT-5 mini/openai_gpt-5-mini-2025-08-07	96.06%	±0.18
Gemini 3 Pro/google_gemini-3-pro-preview 2025 november	96.03%	±0.18
o4-mini/openai_o4-mini-2025-04-16	96.02%	±0.18
Claude Opus 4.5/anthropic_claude-opus-4-5-thinking	95.88%	±0.18

**Table 2:** LRP heatmap from AD patients’ average intensity values, with all values smaller than the 50th percentile of the average AD patient in black, increasing values ranging from red to yellow, and all values greater than the 99.5th percentile in white with multiple β values. Adapted from [163].

Slice 60	Slice 70	Slice 80	Slice 90			
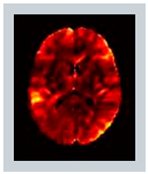	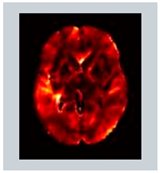	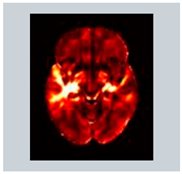	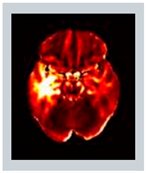	AD	LRP (β = 0)	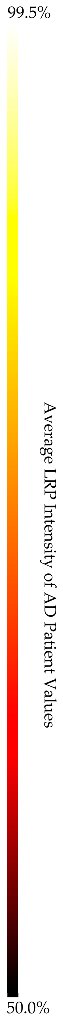
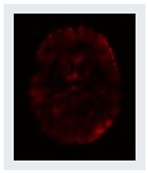	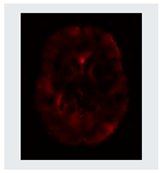	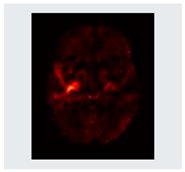	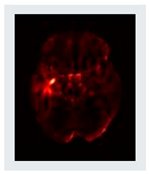	Healthy Control		
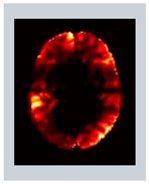	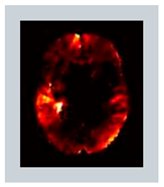	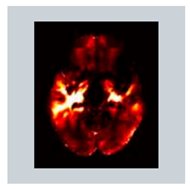	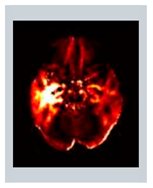	AD	LRP (β = 0.5)	
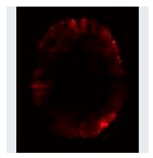	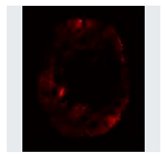	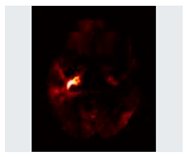	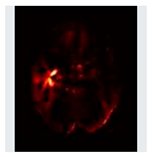	Healthy Control		
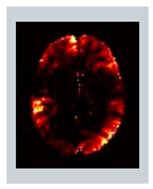	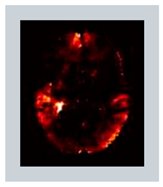	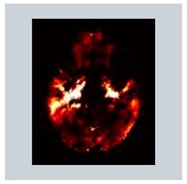	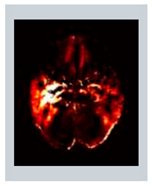	AD	LRP (β = 1)	
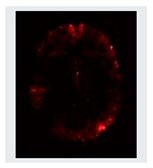	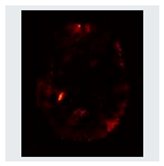	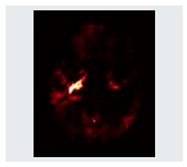	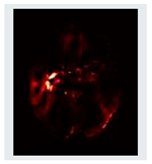	Healthy Control		

**Table 3:** Regulatory requirements and related acceptance criteria.

Article	Regulatory Requirement	Technical Artifact	Acceptance Criterion
Art. 11	Technical Documentation	Model card, system architecture description, explainability report	Documentation enables regulators and notified bodies to understand model design, intended use, limitations, and performance.
Art. 12	Record Keeping and Logging	Audit logs, inference traces, decision logs	Traceability of model outputs and reconstruction of system behavior
Art. 13	Transparency and Provision of Information	User documentation, explanation dashboard, confidence indicators	Users can interpret outputs and understand system capabilities and limitations. Faithfulness, stability, and clinical usefulness metrics must be implemented. Causal relation between explanation and prediction must be established.
Art. 14	Human Oversight	Human-in-the-loop workflow, override mechanisms, alert system	Human operators can monitor, validate, and intervene when necessary.

## Data Availability

This query was used for data extraction and analysis in Figure 1. *(“artificial intelligence” OR AI) AND healthcare* for the AI in Healthcare category, and (“explainable artificial intelligence” OR “explainable AI” OR XAI) AND healthcare for the XAI in healthcare category.

## Abbreviations

The following abbreviations are used in this manuscript:

AD	Alzheimer’s Disease
ADNI	Alzheimer’s Disease Neuroimaging Initiative
AI	Artificial Intelligence
ANOVA	Analysis of Variance
AUC	Area Under the Curve
BMD	Bone Mineral Density
BRAF	B-Raf
CAM	Class Activation Mapping
CE	Conformité Européenne
CGBP	Class Activation Map Guided Backpropagation
CNN	Convolutional Neural Network
CoT	Chain of Thought
CT	Computed Tomography
CXRs	Chest X-rays
CRT-D	Cardiac Resynchronization Therapy Defibrillators
DAG	Directed Acyclic Graph
DBSCAN	Density-Based Spatial Clustering of Applications with Noise
DeepLIFT	Deep Learning Important FeaTures
DNA	Deoxyribonucleic Acid
EHR	Electronic Health Record
EU	European Union
GAM	Generalized Additive Model
GAP	Global Average Pooling
GLM	Generalized Linear Models
GMM	Gaussian Mixture Model
ICA	Independent Component Analysis
ICU	Intensive Care Unit
ICD	Implantable Cardioverter Defibrillator
kNN	k-Nearest Neighbors
LDA	Linear Discriminant Analysis
LIME	Local Interpretable Model-Agnostic Explanations
LLMs	Large Language Models
LRP	Layer-wise Relevance Propagation
LOINC	Logical Observation Identifiers Names and Codes
MCI	Mild Cognitive Impairment
MIMIC	Medical Information Mart for Intensive Care
MIMICSQL	Medical Information Mart for Intensive Care Sequential Query Language
MMLU	Massive Multitask Language Understanding
MRI	Magnetic Resonance Imaging
NIS2	Network and Information Security Directive 2
OCT	Optical Coherence Tomography
OLS	Ordinary Least Squares
PCA	Principal Component Analysis
PET	Positron Emission Tomography
RAGs	Retrieval-Augmented Generation
RectGrad	Rectified Gradient
RISE	Randomized Input Sampling for Explanation
RLHF	Reinforcement Learning from Human Feedback
SAPS II	Simplified Acute Physiology Score
SHAP	Shapley Additive Explanations
SOFA	Sequential Organ Failure Assessment
SNOMED CT	Systematized Nomenclature of Medicine—Clinical Terms
SVM	Support Vector Machine
UCUM	Unified Code for Units of Measure
UCSD	University of California San Diego
UMLS	Unified Medical Language System
US	United States
USMLE	US Medical Licensing Exam
VISTA3D	Versatile Imaging Segmentation and Annotation (model)
WHO	World Health Organization
WSI	Whole-Slide Imaging
XAI	Explainable Artificial Intelligence
